# Chimeric Newcastle Disease Virus Vectors Expressing Human IFN-γ Mediate Target Immune Responses and Enable Multifaceted Treatments

**DOI:** 10.3390/biomedicines11020455

**Published:** 2023-02-04

**Authors:** Rofaida Mostafa Soliman, Keisuke Nishioka, Tomo Daidoji, Osamu Noyori, Takaaki Nakaya

**Affiliations:** 1 Department of Infectious Diseases, Graduate School of Medical Science, Kyoto Prefectural University of Medicine, Kyoto 602-8566, Japan; 2 Department of Animal Medicine, Faculty of Veterinary Medicine, Damanhour University, Damanhour 22511, Egypt; 3 Laboratory of Immunology and Microbiology, College of Pharmaceutical Sciences, Ritsumeikan University, Shiga 525-8577, Japan

**Keywords:** anti-tumor response, chimeric virus, immune response, Newcastle disease virus, recombinant virus vector, vaccination

## Abstract

The therapeutic potential of Newcastle disease virus (NDV) has been reported as both an oncolytic agent and a vaccine vector against many antigens. However, in the individuals already immunized with NDVs, second and subsequent administration does not provide substantial benefits. In this study, two types of recombinant chimeric NDVs using APMV-2 F and HN genes were generated. In rNDV-2HN, the wild-type NDV HN gene was replaced with the APMV-2 HN gene, and in rNDV-2F/2HN, both wild-type F and HN genes were replaced with APMV-2 F and HN genes, respectively. We enhanced the immune responses of these chimeric viruses by inserting the human IFN-γ gene. To examine the escape from NDV antiserum, each virus was treated with diluted NDV antiserum, and HEp-2 cells were infected with these virus particles. The two constructed chimeric viruses indicated notably lower virus-neutralizing titer compared to wild-type NDV and escaped the action of NDV antiserum. These two chimeric viruses infected both respiratory and colon cancer cell lines, indicating their potential as a cancer treatment tool. Chimeric viruses with enhanced immune responses can be considered a novel therapeutic strategy in cancer treatment that can be administered multiple times and used to enhance immune cells interaction.

## 1. Introduction

Newcastle disease is a zoonotic disease caused by the Newcastle disease virus (NDV), and countermeasures must be taken to manage it, especially in poultry farms [1]. Chickens are vaccinated in each poultry farm, because the velogenic strain NDV has a high mortality rate, approximately 90% [2]. On the other hand, NDV is known to be less pathogenic to humans, with symptoms limited to conjunctivitis [3]. Although NDV infects normal cells, they are eliminated by host immune responses; however, NDV can replicate efficiently in cancer cells that indicate deficiency in immune responses [4]. Therefore, it is expected to be a useful tool for cancer treatment in humans. There are several strategies. Several isolated strains of NDV that specifically target cancer cells are administered to cancer patients [5]. Cancer cells are taken from patients and killed by NDV infection, and fragments of these cells are used as cancer vaccines [5]. Recombinant NDV is designed by inserting anti-tumor cytokines and is then administrated to cancer patients [5,6]. Some of the treatment strategies using NDV have been highly effective in cancer treatment [5]. Additionally, because NDV infects a wide range of hosts, including humans, it was investigated as a vaccine vector [7,8]. As mentioned above, NDV is used as a treatment for various purposes. However, once NDV is administered, the host develops immunity, and the effect of the second and subsequent administration is limited [9].

NDV belongs to avian paramyxovirus (APMV) and is also called APMV-1. So far, twenty-two serotypes APMVs have been defined by the International Committee on Taxonomy of Viruses. The pathogenicity to poultry is different between APMVs [3], and NDV (APMV-1) has high mortality [2]. Generally, farm chickens are vaccinated against NDV, and NDV antibodies induce cross reactivity against other APMVs. Therefore, poultry rarely die from APMVs other than NDV. The antigenicity is defined by two surface proteins, F and HN. The APMVs display characteristics that can be replicated in NDV by replacing its surface proteins with those of other APMVs, allowing it to infect and replicate in host cells. NDVs with F and HN genes replaced by APMV-8 genes have already been constructed [10]. Although the NDV-antiserum indicates cross-reaction between these APMVs, its affinity is different. APMV-2, -6, and -10 showed higher ability to escape the NDV antiserum [11]. Therefore, the construction of NDVs with replaced APMV-2 genes was attempted to vaccinated individuals [12,13]. In these reports, the ectodomain regions of F and HN genes were replaced, and they escaped the action of NDV antiserum. Additionally, recombinant NDV with the inserted influenza HA gene also induced protection against avian influenza virus infection in chicken [12,13].

As described previously, recombinant NDVs are useful for cancer therapy and vaccination in poultry, livestock, and human. However, the benefits are limited in individuals who are already immunized by NDVs. Therefore, the user has to choose between different strategies. Here, we introduced the new type chimeric NDVs using APMV-2 F and HN genes. We constructed two chimeric viruses, rNDV-2HN, in which the wild-type NDV HN gene was replaced with the APMV-2 HN gene, and rNDV-2F/2HN, in which both wild-type F and HN genes were replaced with the APMV-2 F and HN genes. We evaluated the immune responses against some cancer cell lines using chimeric NDVs containing the *IFN-γ* gene. We also evaluated the ability of chimeric NDVs containing the *IFN-γ* gene to escape from the NDV antiserum. 

## 2. Materials and Methods

### 2.1. Cell Culture

Chicken embryo fibroblasts (CEFs) were collected from chicken embryo according to a previously described method [14]. Human respiratory epithelial cell lines, HEp-2 and A549 cells, and colon cancer cell line, Caco-2 cells, and chicken fibroblasts, DF-1 cells and CEFs, were cultured and maintained in each medium supplemented with 10% fetal bovine serum (FBS), 100 U/mL penicillin, 100 μg/mL streptomycin, and 250 ng/mL amphotericin B, as previously described [15]. Human peripheral blood mono nuclear cells (PBMC) were isolated from human whole blood using Lymphoprep (Serumwerk Bernburg AG, Bernburg, Germany) and suspended in RPMI1640 medium supplemented with 10% fetal bovine serum (FBS), 100 U/mL penicillin, 100 μg/mL streptomycin, and 250 ng/mL amphotericin B.

### 2.2. Recombinant Chimeric NDV Genome Construction and Rescue Experiment

**Figure 1:**
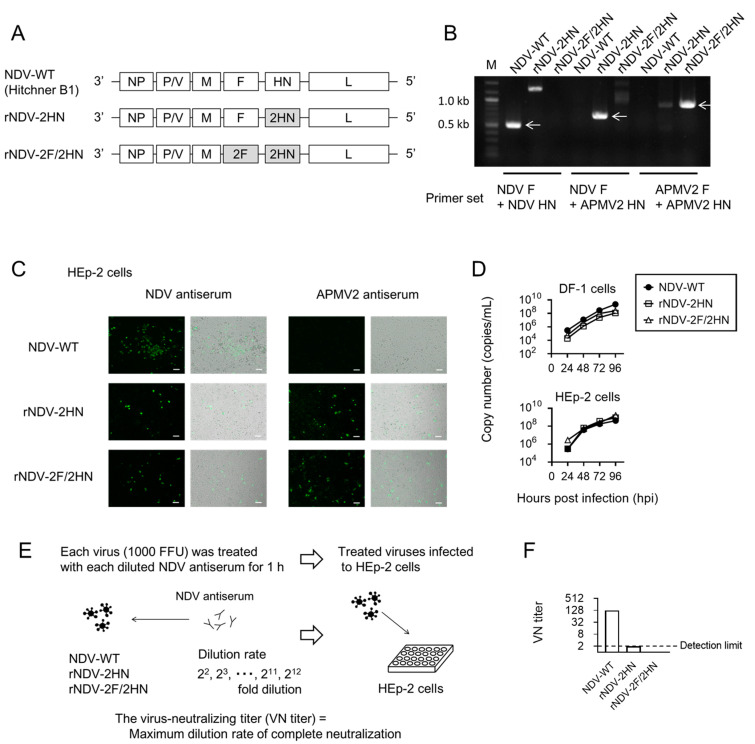

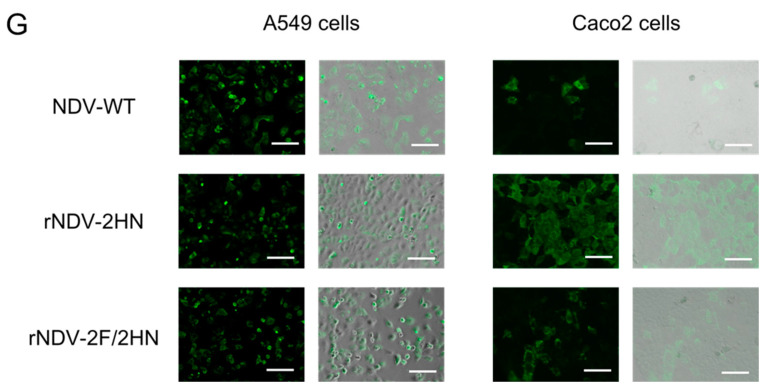
Constructed chimeric NDVs indicated efficient replication and escape from NDV antiserum. (**A**) Structure of each viral genome. In rNDV-2HN, the HN gene was replaced with the APMV-2 HN gene, and in rNDV-2F/2HN, both the F and HN genes were replaced with the APMV-2 F and HN genes, respectively. (**B**) Electrophoresis of PCR products, containing amplified cDNA derived from viral genomes using each primer set. Arrows indicate expected size. Cells were infected with each recombinant virus at an MOI of 0.5 (**C**,**G**) or 0.1 (**D**). (**C**) Infected HEp-2 cells were stained with NDV or APMV-2 antisera. Scale bar, 50 µm. (**D**) Viral replication was assessed in DF-1 (chicken cell line) and HEp-2 cells (human cell line). After infection, the supernatants were harvested at the indicated time points and the copy number of each virus genome was estimated using real-time PCR (*n* = 3). (**E**) Schema of neutralization assay. (**F**) VN titer of each recombinant NDV (*n* = 3). (**G**) The cells infected with each virus were stained by NDV antiserum. Scale bar, 50 µm. Data represent the mean ± SEM.

The Hitchner B1 strain of NDV was used, which is one of the vaccine strains. This genome was already inserted into the pSL1180 vector [16,17]. APMV-2 F and HN genes were amplified according to the APMV-2 genome sequence (Accession no. LC187305). Using an In-Fusion HD Cloning kit (Takara Bio USA, Inc., San Jose, CA, USA), these NDV genes were replaced. Constructed plasmids were confirmed by PCR (Figure S1) using each primer set (shown in Table S1) and sequencing. Finally, we obtained four types of viral genomes and used three as follows; NDV-WT (Hitchner B1), rNDV-2HN, which replaced the HN gene, and rNDV-2F/2HN, which replaced both the F and HN genes. The genomes were cloned into the pSL1180 vector (Figure 1A).

**Figure 2:**
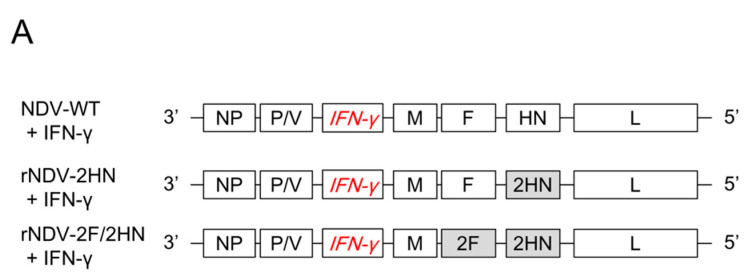

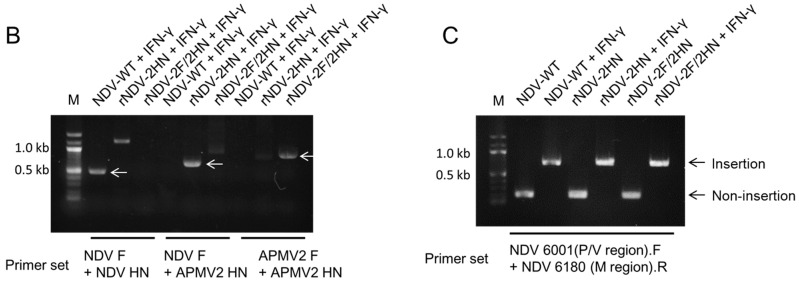
IFN-γ was inserted into all recombinant NDVs. (**A**) Structure of each viral genome. IFN-γ was inserted between the P/V and M genes. (**B**,**C**) Electrophoresis of PCR products, which contain amplified cDNA derived from viral genomes using each primer set. Arrows indicate expected size.

The *IFN-γ* gene was amplified by PCR according to sequence (Accession no. NM_000619) and inserted between the P/V and M genes using an In-Fusion HD Cloning kit. All construct structures are indicated in Figure 2A. Infectious viral particles were obtained through rescue experiments, as previously described [17]. 

Viral propagation was conducted using embryonated eggs, and the allantoic fluid was harvested and purified by ultracentrifugation with 20% sucrose cushion for 2.5 h at 26,000 rpm at 4 °C. Total RNA was extracted from each purified viral solution using ISOGEN II (Nippon Gene, Tokyo, Japan), and reverse transcription was performed by ReverTra Ace qPCR RT Master Mix (TOYOBO, Osaka, Japan). Unique amplification patterns were confirmed by PCR (Figure 1B and Figure 2B,C) using the primers, as shown in Table S1. Titers were determined by fluorescent focus unit (FFU) using the immunostaining assay, according to previously described methods [15]. Pathogenicity was evaluated by mean death time (MDT) in embryonated chicken eggs, according to previously described methods [18]. Viral genome stability was confirmed by sequencing the F and HN genes using virus passaged in embryonated eggs and virus passaged in HEp-2 cells. Experiments using viruses were conducted at the Kyoto Prefectural University of Medicine under Biosafety Level 2 conditions. All experiments using recombinant DNA were conducted in accordance with the relevant Japanese laws. Risk assessments were conducted by the Living Modified Organisms Committee of Kyoto Prefectural University of Medicine and by the Ministry of Education, Culture, Sports, Science, and Technology of Japan. After these assessments, the experiments and modified methods were approved by the Biological Safety Committee of Kyoto Prefectural University of Medicine (Approval number 2022-113, approved 26 October 2018 and 24 June 2022).

### 2.3. Immunofluorescence Assay

The cells were cultured in 24-well plate (1.0 × 10^5^ cells/well) and then infected with the viruses at an MOI of 0.5, according to the influenza virus infection method [15]. Immunofluorescence assay was performed according to the previously described method [15]. Twenty-four hours after infection, the cells were fixed with PBS containing 4% paraformaldehyde for 10 min at room temperature, and permeabilized with 0.2% Triton X-100 in PBS for 10 min at room temperature. Chicken anti-NDV and chicken anti-APMV-2 antisera or rabbit anti-IFN-γ IgG polyclonal antibody (cat no. 15365-1-AP, Proteintech, Tokyo, Japan) were used as primary antibodies. Secondary antibody treatment was performed using goat anti-Chicken IgY conjugated Alexa-488 (cat no. A11039, ThermoFisher Scientific, Waltham, MA, USA) or anti-Rabbit IgG conjugated Alexa-555 (cat no. A21428, ThermoFisher Scientific, Waltham, MA, USA), respectively. Infected cells were observed by fluorescence microscopy BZ-X700 (KEYENCE, Osaka, Japan). 

### 2.4. Viral Replication, Neutralization, and IFN-γ RNA Expression Assays

DF-1, HEp-2, and A549 cells were cultured in 24-well plates (2.0 × 10^5^ cells/well) and infected with the recombinant NDVs at a multiplicity of infection (MOI) of 0.1. The supernatant was harvested at 24, 48, 72, and 96 h post infection. After RNA extraction and reverse transcription, the viral copy number was estimated by real time PCR using PowerUp SYBR Green Master Mix (Thermo Fisher Scientific, Waltham, MA, USA), and the following forward (5′-AGTGATGTGCTCGGGCCTTC-3′) and reverse (5′-CCTGAGGAGAAGCATTTGCTA-3′) primers.

In the neutralization assay, chicken anti-NDV antiserum was serially diluted 2-fold and prepared from 1st power of 2 to the 12th power of 2 diluted antisera, as shown in Figure 1E. Each viral solution (1000 FFU) was infected with HEp-2 cells 1 h after treatment with each concentration of the antisera. Twenty-four hours after infection, the infected cells were assessed using immunofluorescence. The maximum dilution rate of complete neutralization was determined as the virus-neutralizing titer (VN titer). 

HEp-2 cells cultured in 24-well plates (2.0 × 10^5^ cells/well) were infected with the recombinant NDVs at an MOI of 1. Twenty-four hours after infection, total RNA was extracted from the infected cells and then reverse transcription was performed. *IFN-γ* RNA expression was evaluated by real time PCR ^ΔΔ^Ct method using forward (5′-TCGGTAACTGACTTGAATGT-3′) and reverse (5′-TCGCTTCCCTGTTTTAGCTG -3′) primers. 

### 2.5. Cytotoxicity Assay

HEp-2 and A549 cells were cultured in 96-well plates (5.0 × 10^3^ cells/well) and infected with the recombinant NDVs at an MOI of 0.01, 0.1, or 1. The live cells were evaluated by MTT assay using Cell Count Reagent SF (Nacalai Tesque, Kyoto, Japan) at 24, 48, and 72 h post infection. Cell viability was calculated based on mock infection at each indicated time point.

### 2.6. Enzyme-Linked Immunosorbent Assay (ELISA)

For evaluating the IFN-γ protein expression level, ELISA was performed (cat. no. DY285B-05; R&D Systems, Minneapolis, MN, USA). Supernatants harvested at 24, 48, and 72 h post infection by each virus were used as a sample.

### 2.7. Killing Assay

The target cells, HEp-2 and A549 cells, were cultured in 96-well plates (1.0 × 10^4^ cells/well) and infected with the recombinant NDVs at an MOI of 0.2. After washing twice with PBS, human PBMC was added to the plate as effector cells. Ratio of the effector cells to target cells (E:T ratio) were prepared in four conditions: 0:1, 1.25:1, 2.5:1, and 5:1. Killing assay was also performed in the presence of NDV antiserum. Each recombinant NDV was mixed with 64-fold-diluted antiserum. After 1 h, the recombinant NDVs and PBMC were added to the plate. The MOI and E:T ratio were kept constant. After 24 h, cell viability was evaluated. 

### 2.8. Statistical Analysis

Data are expressed as mean ± SEM. All statistical analyses were performed by Student’s *t*-tests using GraphPad Prism 8 software (La Jolla, CA, USA) *p* values of < 0.05 were considered statistically significant.

## 3. Results

### 3.1. Two Types of Infectious Chimeric NDVs Were Constructed

In Figure 1, chimeric NDVs construction and the properties were confirmed. We constructed two types of infectious chimeric NDVs, namely rNDV-2HN, which replaced the wild-type NDV (NDV-WT) HN gene with the APMV-2 HN gene, and rNDV-2F/2HN, which replaced the NDV-WT F and HN genes with the APMV-2 F and HN genes, respectively (Figure 1A). To confirm the recombination of these genes, PCR was performed using primers designed at each gene and cDNA that was reverse-transcribed from the RNA of each recombinant virus (Figure 1B). Only in the case of specific cDNAs and primers were strong bands observed at the target size, as indicated by arrows. Although some non-specific bands that indicated lower intensity were observed, they were also observed in the PCR using the constructed plasmids as a template (Figure S1). To investigate the stability of the chimeric NDV genome, the F and HN genes of the virus passaged in embryonated eggs and HEp-2 cells were sequenced (Figure S2). Viruses propagated in embryonated eggs indicated only one base mutation in the F of both chimeric NDVs and no mutations in the HN genes. Amino acid mutation was detected in the F of rNDV-2F/2HN (aa: I104L in ectodomain). Viruses propagated in HEp-2 cells at least three times only showed a few mutations (Figure S2B). Therefore, each constructed virus particle has recombinant genes and presents high stability. Subsequently, infectivity and antigen expression were confirmed by immunostaining using NDV and APMV-2 antisera. HEp-2 cells, which were respiratory epithelial cell lines infected with NDV-WT, were stained by NDV antiserum, whereas the cells infected with rNDV-2HN and rNDV-2F/2HN were stained by both NDV and APMV-2 antisera (Figure 1C). Infectious viruses expressing the targeted antigen were identified. rNDV-2HN was able to infect the cells using different viral surface proteins. The cells infected with rNDV-2HN and rNDV-2F/2HN expressed both NDV and APMV-2 antigens. Viral replication rate in the chicken (DF-1 cells) and human cell lines (HEp-2 cells) was also evaluated. Viral replication was shown in both cell lines, and its replication rate was similar among these recombinant NDVs (Figure 1D). To confirm the escape from NDV antiserum, each virus was treated with diluted NDV antiserum, following which HEp-2 cells were infected with the virus (Figure 1E). The two types of chimeric viruses showed lower values of virus-neutralizing titer (VN titer) and escape from NDV antiserum (Figure 1F). HN is a major antigen that has the possibility of inducing NDV-neutralizing antibodies during infection [19,20]. Replacement of HN resulted in a significant escape from antisera. Some human cancer cell lines were infected to explore the potential of these chimeric viruses as cancer treatment tools. These two types of chimeric virus indicated infection to both respiratory (A549) and colon cancer (Caco2) cell lines, and showed potential as a cancer treatment tool similar to NDV-WT (Figure 1G). 

### 3.2. Recombinant NDVs with Inserted Human IFN-γ Gene Were Constructed and Indicated Low Pathogenicity

To use as a cancer treatment tool, recombinant (also chimeric) NDVs were constructed by inserting the human *IFN-γ* gene between the P/V and M genes (Figure 2A). As shown in Figure 2B,C, the human *IFN-γ* gene could be inserted into these viruses, having different combinations of F and HN genes, using PCR. cDNA that was reverse-transcribed from the RNA of each recombinant virus and relevant primer sets were used. For immunostaining after HEp-2 cell infection, the cells infected with recombinant NDVs inserted with the *IFN-γ* gene were stained by anti-IFN-γ antibody (Figure S3). IFN-γ expression in the infected cells was confirmed. Mean death time in the embryonated eggs indicated more than 168 h in all recombinant NDVs (Table 1). Although there may be changes in pathogenicity due to genetic recombination, all recombinant NDVs indicated much lower pathogenicity in the embryonated eggs.

### 3.3. Recombinant NDVs + IFN-γ Induced production of Sufficient IFN-γ in the Infected HEp-2 Cells

**Figure 3:**
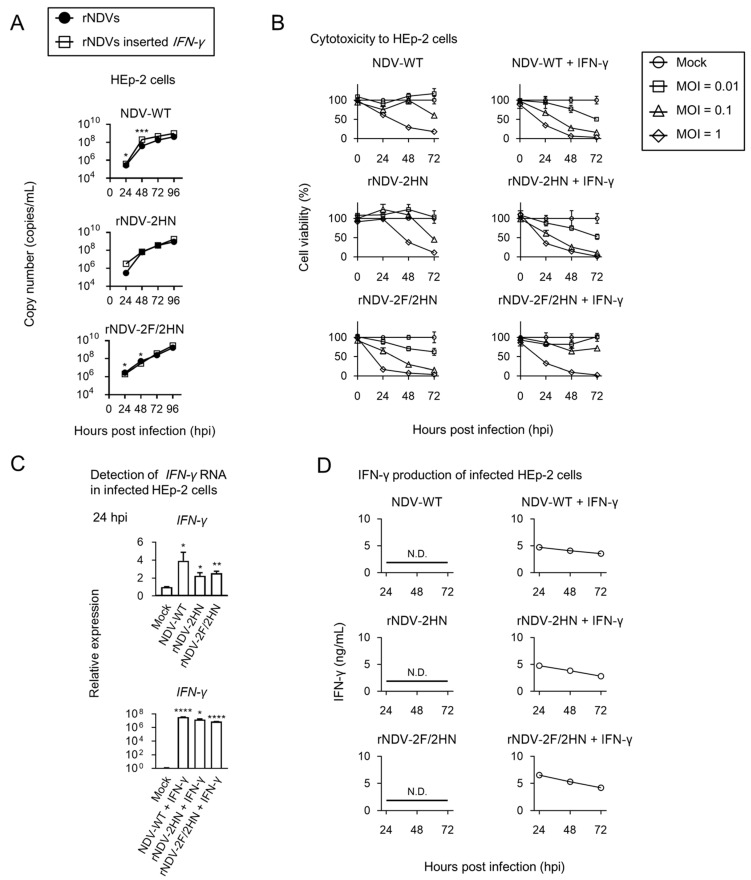
Insertion of *INF-γ* gene induced change in cytotoxicity and sufficient IFN-γ production. HEp-2 cells were infected with each recombinant virus at an MOI of 0.1 (**A**,**C**,**D**) or 0.01, 0.1, and 1 (**B**). (**A**) Viral replication was assessed. After infection, the supernatants were harvested at the indicated time points. The copy number of each virus genome was estimated using real-time PCR and compared to that of viruses without *IFN-γ* insertion (*n* = 3, * *p* < 0.05, *** *p* < 0.001). (**B**) Cytotoxicity was evaluated by MTT assay at 0, 24, 48, and 72 h post infection. (**C**) Total *IFN-γ* RNA was detected in infected cells 24 h after infection. Expression levels compared to mock infection (*n* = 3, * *p* < 0.05, ** *p* < 0.01, **** *p* < 0.0001). (**D**) Evaluation of IFN-γ production in the supernatants was evaluated by ELISA at the indicating time points (*n* = 3). Data represent the mean ± SEM.

Next, replication rate, cytotoxicity, *IFN-γ* gene expression, and IFN-γ production of constructed recombinant NDVs were evaluated in human cell lines (Figure 3). Replication rate was slightly increased in NDV-WT with the IFN-γ gene, compared to NDV-WT. Other viruses indicated similar replication rates (Figure 3A). Cytotoxicity to HEp-2 cells was changed by inserting *IFN-γ*; the viability of cells was lower when infected with NDV-WT + IFN-γ and rNDV-2HN + IFN-γ, and higher when infected with rNDV-2F/2HN + IFN-γ, compared to the viability observed under no *IFN-γ* insertion (Figure 3B). Although IFN-γ mainly induces inflammation via activation of immune cells, this cytokine also induces antiviral responses to surrounding cells that are much weaker than type I interferon. Because immune cells were not used in this experiment, it was expected that *IFN-γ* insertion would result in lower cytotoxicity. However, no major changes were observed in any case. It was shown that rNDVs + IFN-γ exhibited parental cytotoxicity against some cancer cells, even if IFN-γ was present. To evaluate whether infected cells produced sufficient IFN-γ, RNA and protein expression levels were evaluated. *IFN-γ* RNA was found to be induced, not only upon infection with recombinant NDVs + IFN-γ, but also in cells infected with viral cells without IFN-γ gene insertion (Figure 3C). However, the levels induced by recombinant NDVs without IFN-γ gene insertion were strikingly lower compared to that induced by recombinant NDVs + IFN-γ. Consistent with the results of RNA induction, IFN-γ production was only detected in the cells infected with recombinant NDVs + IFN-γ (Figure 3D). IFN-γ produced by infected cells may induce anti-tumor responses, because this concentration (approximately 5 ng/mL) was generally used in immunology experiments.

### 3.4. Recombinant NDVs + IFN-γ Lead to Anti-tumor Responses of PBMC

**Figure 4:**
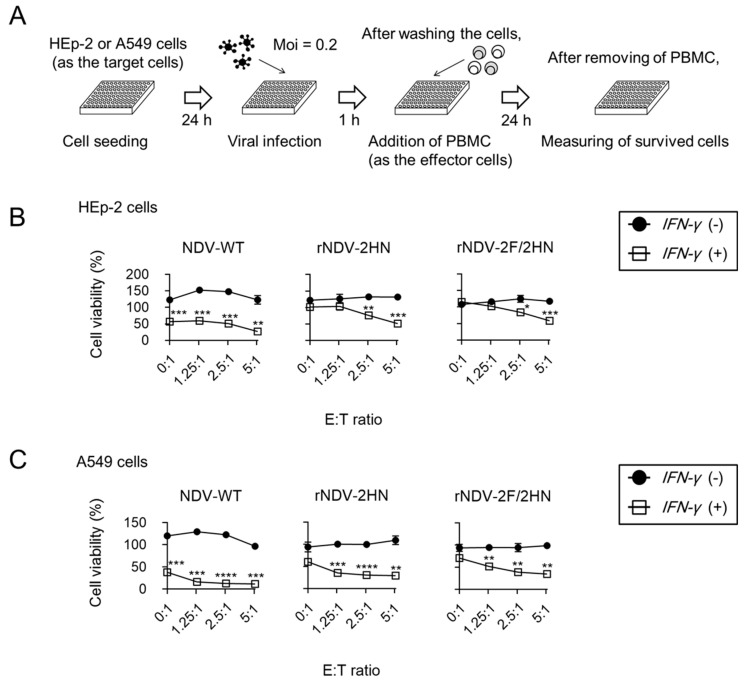
Recombinant NDVs + IFN-γ induced modification of anti-tumor responses by PBMC. (**A**) Schema of killing assay using PBMC. (**B**,**C**) Cell viability of HEp-2 and A549 cells 24 h after infection at each E:T ratio. Cell viability was compared to that observed when infected with viruses without *IFN-γ* insertion (*n* = 3, * *p* < 0.05, ** *p* < 0.01, *** *p* < 0.001, **** *p* < 0.0001).

Finally, we assessed whether the infection of recombinant NDVs + IFN-γ modified immune responses around the infected cells. IFN-γ is one of the important cytokines in antitumor response, with functions such as repressing tumor cell proliferation, increasing antigen presenting function from dendritic cells, and enhancing the killer activity of cytotoxic T lymphocytes [21]. Therefore, killing assay was performed using human PBMCs, including T cells, B cells, natural killer cells, dendritic cells, and monocytes as the effector cells (Figure 4A). Compared to mock infection, after adding the effector cells, cell viability was decreased in a dose dependent manner in the two human cell lines, HEP-2 (Figure 4B) and A549 (Figure 4C), which were infected with recombinant NDVs + IFN-γ. Moreover, the cells infected with recombinant NDVs without *IFN-γ* insertion did not show changes in cell viability upon adding PBMC. In this experiment, the medium was changed after PBMC addition to evaluate the function of PBMC (Figure S4A). Because the cell viability without PBMC addition (E:T = 0:1) was different than that in Figure 3B, the cell viability of HEp-2 cells was evaluated under the same medium conditions (Figure S4B). The growth rate of HEp-2 cells when cultured in RPMI1640 medium was reduced as compared to DMEM cultivation (Figure S4C). Therefore, the differences in cell viability may be because the growth rate of host cells affected viral replication. However, in this experiment, it is important to improve the anti-tumor response of PBMC to cells infected with recombinant NDV + IFN-γ. Infection with recombinant NDVs + IFN-γ was cytotoxic to the cancer cells (Figure 3B), and also improved the anti-tumor responses of immune cells. 

### 3.5. Infection of rNDV-2HN and 2F/2HN + IFN-γ Induced Anti-tumor Responses in the Presence of Anti-NDV Antiserum

**Figure 5:**
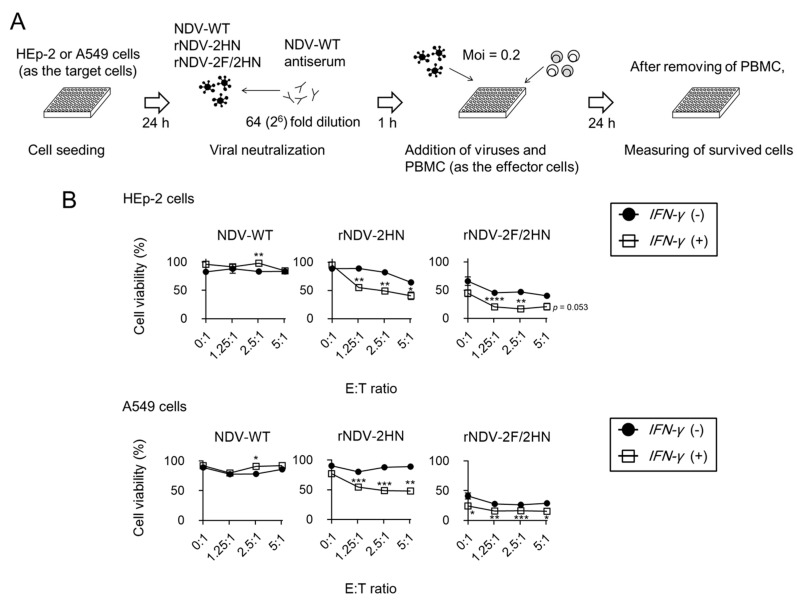
In the presence of NDV antiserum, rNDV-2HN/rNDV-2F2HN + IFN-γ induced modification of anti-tumor responses by PBMC. (**A**) Schema of killing assay using PBMC in the presence of NDV antiserum. (**B**) Cell viability of HEp-2 and A549 cells 24 h after infection at each E:T ratio. Cell viability was compared to that observed when infected with viruses without *IFN-γ* insertion (*n* = 3, * *p* < 0.05, ** *p* < 0.01, *** *p* < 0.001, **** *p* < 0.0001).

Killing assay was also used in the NDV antiserum condition (Figure 5A). Upon infection with NDV-WT and NDV-WT + IFN-γ, the cell viability was almost 100% compared to mock infection. Consistent with previous reports, these viruses were hard to infect in the presence of NDV antiserum. On the other hand, rNDV-2HN and rNDV-2F/2HN with and without IFN-γ insertion were able to infect target cells in this condition due to decreased cell viability (Figure 5B,C). Additionally, infection with rNDV-2HN and rNDV-2F/2HN + IFN-γ also induced anti-tumor responses in a dose-dependent manner. When the E:T ratio was 0:1, the cell viability was significantly different from that shown in Figure 4. This could be because the NDV antiserum, which included various factors, was present during the steps shown in Figure 5. From these results, we conclude that immunized individuals could possibly receive multiple treatments, including cancer treatment, using these recombinant NDVs.

## 4. Discussion

When the surface proteins of NDV are replaced with other APMV surface proteins, viral particles can still replicate in host cells. Although pseudo-type viruses, such as those based on lentiviruses [22] and vesicular stomatitis virus [23], often express the surface antigens of other viruses, replacement with other antigens is rare, even if mutations are introduced through rescue experiments. For example, rescue experiments have been used to construct the influenza virus, in which some genes are replaced with other types of influenza virus antigens. However, in the influenza virus, when a different type of virus infects the same individual, reassortment occurs, that is, the genomes of eight segments are mixed, and a new type of virus is formed naturally, which contains a mixture of these genomes [24]. However, NDVs have one segment genome, and reassortment does not occur. Therefore, this property is a rare feature, and we can construct viruses that are difficult to obtain naturally. Many studies have investigated the use of NDV as an oncolytic agent against different kinds of cancers, such as gastric cancer and melanoma, and have reported the use of reverse genetics to enhance the oncolytic properties of NDV [25]. NDV is able to target cancer cells owing to the defective interferon pathways within tumor cells that are not present in normal cells [4]. Therefore, normal cells do not allow NDV to replicate efficiently, making it a safe vector for clinical use. Here, we constructed two new types of chimeric viruses, rNDV-2HN and rNDV-2F/2HN. In particular, rNDV-2HN was constructed by combining different types of surface proteins (NDV F and APMV-2 HN); it also escaped from the NDV antiserum. Constructing viruses by combining the surface antigens of different viral species could potentially produce numerous chimeric virus constructs using all reported APMVs. This may be very useful for multiple treatments in patients.

Kim et al. [26] presented an important report on the construction of chimeric NDVs, in which they attempted to construct chimeric NDVs and APMV-2 by replacing F and HN genes with their APMV-2 or NDV counterparts, respectively. For example, NDV-replaced APMV-2 F and/or HN was not recovered, and chimeric viruses were only recovered when the ectodomains of the NDV, APMV-2 F, and HN genes were replaced. To discuss why our results differ from those in previous reports, we analyzed the identity of the F and HN proteins among the investigated virus strains. As shown in Figure S2C, although the differences were equivalent to a few percent overall, the transmembrane and endodomain regions indicated high identities. In particular, because the transmembrane and endodomain regions of APMV2 HN presented 100% identity, the differences in these regions did not determine viral construction. The previous study [26] concluded that the APMV-2 spike was not compatible with NDV internal components. However, this study suggests that the spikes and internal components of NDV and APMV-2 are not incompatible and that differences in tropism between NDV strains may have an effect. The previous study that constructed a chimeric virus in which the APMV-2 F gene was replaced with NDV [26] supports this consideration. Further research is required for the construction of many chimeric viruses.

In the current study, human IFN-γ, also known as immune interferon, a cytokine with multiple functions, which acts on almost all immune cells and both innate and adaptive immune responses, was inserted into recombinant NDVs, including chimeric viruses, to assess cancer treatment candidates. It is well known that IFN-γ increases antigen presentation by macrophages and activates antigen presenting cells, promoting Th1 differentiation and suppressing Th2 cell activity. Moreover, IFN-γ has direct antitumor and anti-angiogenic effects, inhibiting proliferation and sensitizing the tumor cells to apoptosis [21]. The cells infected with recombinant NDVs + IFN-γ produced sufficient IFN-γ to modify immune responses and enhanced the killing activities of human PBMC. Additionally, rNDV-2HN and -2F/2HN showed similar functions, even when presented with NDV antiserum. These results suggest the possibility that recombinant NDVs + IFN-γ can modify immune responses, at least around infected cells, in cancer patients, and that chimeric viruses can be used for cancer treatment. Already, NDV-inserted human IL-2 and inserted human IL-12 have been constructed [6,27,28]. Additionally, in murine genes, IFN-γ, IL-2, and TNF-α insertions to NDV were already confirmed [29]. In this report, because murine IFN-γ effects were weaker than other cytokines, the effects of human IFN-γ were not confirmed. However, in the current study, we confirmed human IFN-γ activities against some cancer cell lines. Therefore, the combination of these treatments is considered to be more effective for cancer treatment. In this study, we evaluated the infection of chimeric viruses in respiratory and colon cancer cell lines. Oncolytic NDV has been used in several cancer treatments, including colorectal cancer, ovarian cancer, melanoma, and renal cell carcinoma [5]. Other types of cells can also be infected, and confirmation of infection may lead to a wide range of applications, such as NDV. Meanwhile, NDVs are also used to vaccinate poultry and livestock [7]. Insertion genes were mainly targeted in the antigens of pathogens because treatment options were limited. However, these chimeric NDVs suggest that enhancing host immune responses by inserting cytokine genes is a novel strategy. Using this strategy, higher levels of antibody induction, immune cell interaction, and recruitment of target immune responses can be expected. The immune response induced by NDV infection in healthy individuals must be thoroughly investigated for this purpose; however, NDV has various possibilities, as it has a wide range of hosts, including human. The antitumor activity of NDV expressing other cytokines has been evaluated using mice [27,28]. These animal models allow for the evaluation of not only the effect of antitumor responses but also the effect of the chimeric NDV combination on vaccine evaluation, such as the combination of NDV with an inserted target antigen and chimeric NDV with an inserted cytokine gene.

Additionally, because the effect on normal cells can be evaluated, it is possible that chimeric NDVs can be applied for various uses. Therefore, further studies need to investigate the use of the constructed chimeric viruses with inserted human IFN-γ in animal models. 

## 5. Conclusions

Recombinant NDVs with inserted *IFN-γ* were found to modify immune responses around the infected cells and reduce the cell viability of cancer cells upon adding PBMC cells. The current study is expected to provide an important basis for further research and application of chimeric NDV in cancer immunotherapy regarding using it for multiple treatments. In particular, the construction of chimeric NDV by combining surface antigens of different viral species indicates the possibility of constructing many types.

## Figures and Tables

**Table 1:** Mean death time in embryonated eggs.

Virus	Mean Death Time
NDV-WT	>168 h
NDV-WT + IFN-γ	>168 h
rNDV-2HN	>168 h
rNDV-2HN + IFN-γ	>168 h
rNDV-2F/2HN	>168 h
rNDV-2F/2HN + IFN-γ	>168 h

Velogenic strains, <60 h; mesogenic strains, 60 to 90 h; lentogenic strains, >90 h.

## Data Availability

Not applicable.

## Supplementary Materials

The following supporting information can be downloaded at: https://www.mdpi.com/article/10.3390/biomedicines11020455/s1, Figure S1: Electrophoresis indicated higher intensity specific band and lower intensity non-specific bands using plasmids; Figure S2: Analysis of chimeric NDVs gnome stability; Figure S3: INF-γ expression was detected in infected cells with recombinant NDVs + IFN-γ; Figure S4: HEp-2 cell proliferation was modified by RPMI1640 medium; Table S1: Primer sets used in this study.
