# Supplementary material for: Chimeric Newcastle Disease Virus Vectors Expressing Human IFN-γ Mediate Target Immune Responses and Enable Multifaceted Treatments
Source: Biomedicines. 2023 Feb 4;11(2):455. doi: 10.3390/biomedicines11020455 (PMC9953603; doi:10.3390/biomedicines11020455)
Supplement: Supplementary file 1 [file biomedicines-11-00455-s001.zip › biomedicines-2159350-supplementary.pdf]

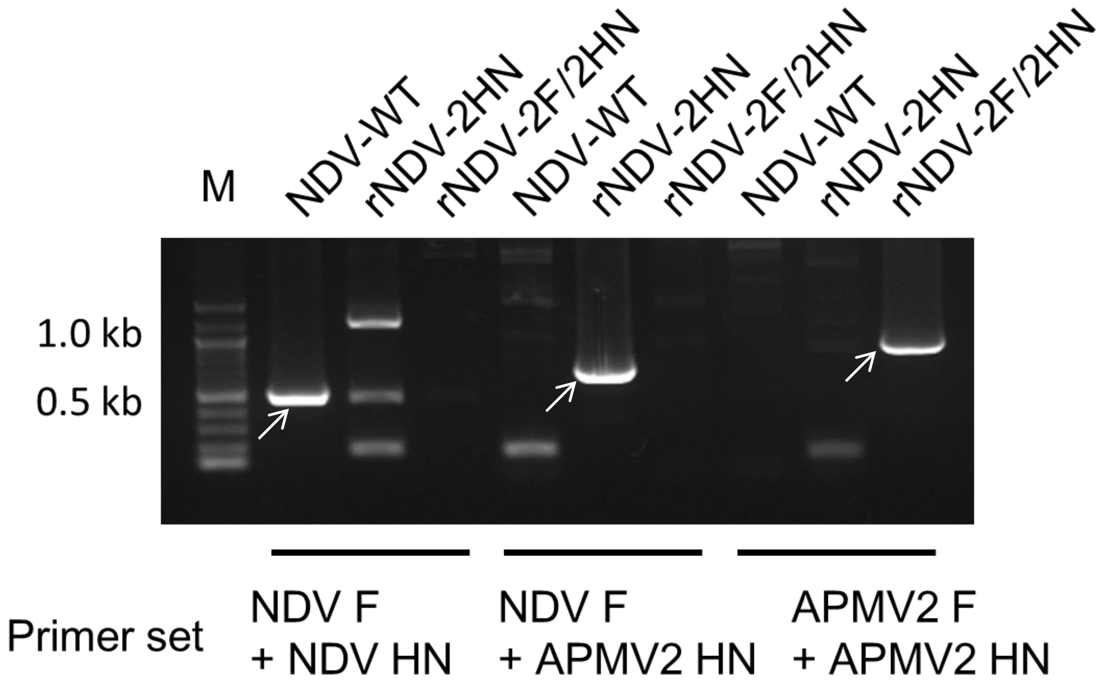

Figure S1. Electrophoresis indicated higher intensity specific band and lower intensity non-specific bands using plasmids.
Electrophoresis of PCR which amplified plasmid inserted in each viral genome using each primer set. Arrows indicate expected size.

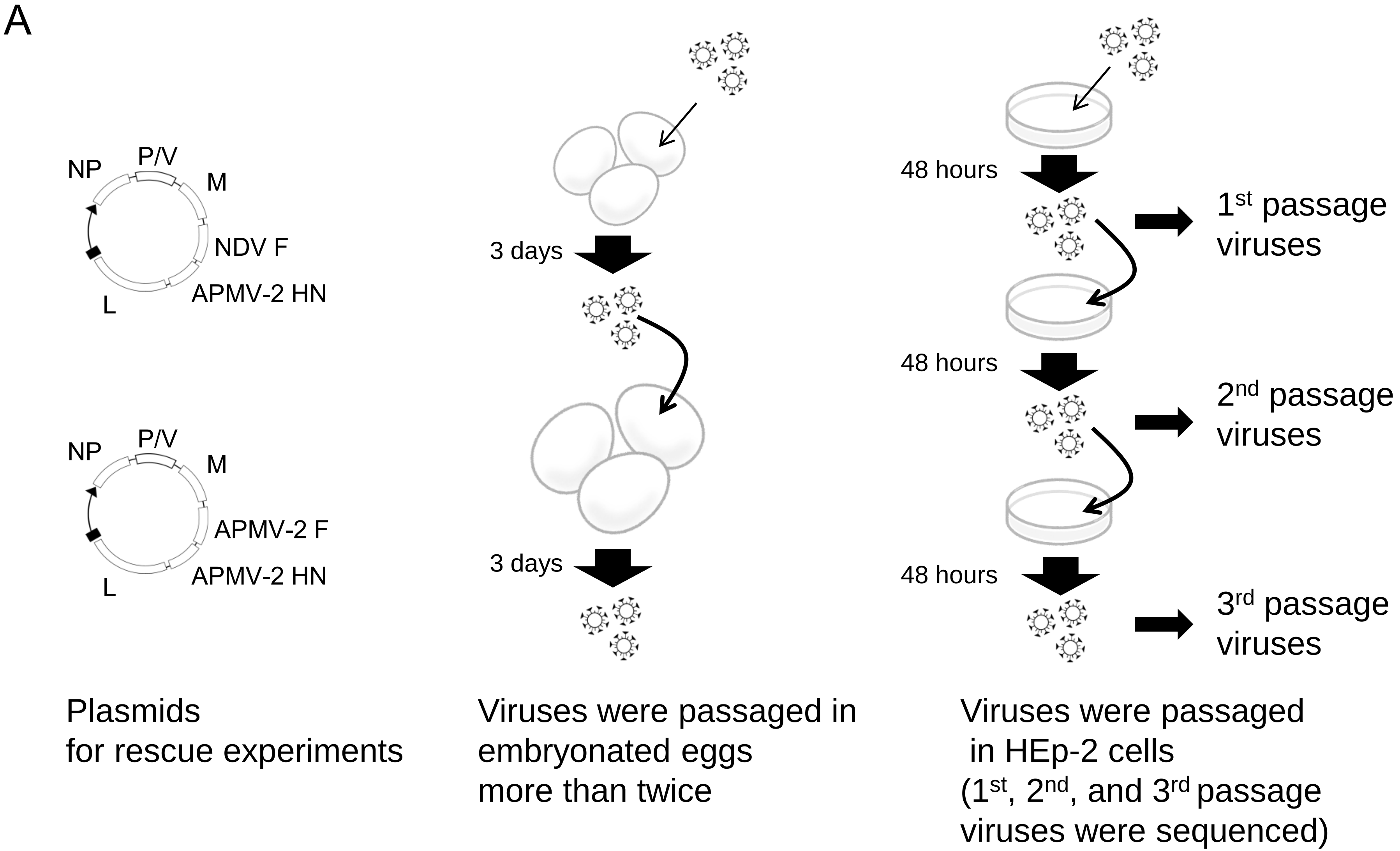
A
NP
P/V
M
NDV F
APMV-2 HN
L
NP
P/V
M
APMV-2 F
APMV-2 HN
L
Plasmids
for rescue experiments
3 days
3 days
Viruses were passaged in
embryonated eggs
more than twice
48 hours
48 hours
48 hours
1st passage
viruses
2nd passage
viruses
3rd passage
viruses
Viruses were passaged
in HEp-2 cells
(1st, 2nd, and 3rd passage
viruses were sequenced)

B

Plasmid : Plasmids for rescue experiments
Egg : Viruses were propagated in embryonated eggs
1st Cell :
2nd Cell : Each passage viruses in HEp-2 cells
3rd Cell :

rNDV-2HN (NDV F gene)

```
Plasmid      1 ATGGGCTCCAGACCTTCTACCAAGAACCCAGCACCTATGATGCTGACTATCCGGGTCGCGCTGGTACTGAGTTGCATCTGCCCGGCAAACTCCATTGATGGCAGGCCTCTTGCAGCTGCA 120
Egg          1 ATGGGCTCCAGACCTTCTACCAAGAACCCAGCACCTATGATGCTGACTATCCGGGTCGCGCTGGTACTGAGTTGCATCTGTCCGGCAAACTCCATTGATGGCAGGCCTCTTGCAGCTGCA 120
1st Cell     1 ATGGGCTCCAGACCTTCTACCAAGAACCCAGCACCTATGATGCTGACTATCCGGGTCGCGCTGGTACTGAGTTGCATCTGTCCGGCAAACTCCATTGATGGCAGGCCTCTTGCAGCTGCA 120
2nd Cell     1 ATGGGCTCCAGACCTTCTACCAAGAACCCAGCACCTATGATGCTGACTATCCGGGTCGCGCTGGTACTGAGTTGCATCTGTCCGGCAAACTCCATTGATGGCAGGCCTCTTGCAGCTGCA 120
3rd Cell     1 ATGGGCTCCAGACCTTCTACCAAGAACCCAGCACCTATGATGCTGACTATCCGGGTCGCGCTGGTACTGAGTTGCATCTGTCCGGCAAACTCCATTGATGGCAGGCCTCTTGCAGCTGCA 120

Plasmid    121 GGAATTGTGGTTACAGGAGACAAAGCAGTCAACATATACACCTCATCCCAGACAGGATCAATCATAGTTAAGCTCCTCCCGAATCTGCCCAAGGATAAGGAGGCATGTGCGAAAGCCCCC 240
Egg        121 GGAATTGTGGTTACAGGAGACAAAGCAGTCAACATATACACCTCATCCCAGACAGGATCAATCATAGTTAAGCTCCTCCCGAATCTGCCCAAGGATAAGGAGGCATGTGCGAAAGCCCCC 240
1st Cell   121 GGAATTGTGGTTACAGGAGACAAAGCAGTCAACATATACACCTCATCCCAGACAGGATCAATCATAGTTAAGCTCCTCCCGAATCTGCCCAAGGATAAGGAGGCATGTGCGAAAGCCCCC 240
2nd Cell   121 GGAATTGTGGTTACAGGAGACAAAGCAGTCAACATATACACCTCATCCCAGACAGGATCAATCATAGTTAAGCTCCTCCCGAATCTGCCCAAGGATAAGGAGGCATGTGCGAAAGCCCCC 240
3rd Cell   121 GGAATTGTGGTTACAGGAGACAAAGCAGTCAACATATACACCTCATCCCAGACAGGATCAATCATAGTTAAGCTCCTCCCGAATCTGCCCAAGGATAAGGAGGCATGTGCGAAAGCCCCC 240

Plasmid    241 TTGGATGCATACAACAGGACATTGACCACTTTGCTCACCCCCCTTGGTGACTCTATCCGTAGGATACAAGAGTCTGTGACTACATCTGGAGGGGGGAGACAGGGGCGCCTTATAGGCGCC 360
Egg        241 TTGGATGCATACAACAGGACATTGACCACTTTGCTCACCCCCCTTGGTGACTCTATCCGTAGGATACAAGAGTCTGTGACTACATCTGGAGGGGGGAGACAGGGGCGCCTTATAGGCGCC 360
1st Cell   241 TTGGATGCATACAACAGGACATTGACCACTTTGCTCACCCCCCTTGGTGACTCTATCCGTAGGATACAAGAGTCTGTGACTACATCTGGAGGGGGGAGACAGGGGCGCCTTATAGGCGCC 360
2nd Cell   241 TTGGATGCATACAACAGGACATTGACCACTTTGCTCACCCCCCTTGGTGACTCTATCCGTAGGATACAAGAGTCTGTGACTACATCTGGAGGGGGGAGACAGGGGCGCCTTATAGGCGCC 360
3rd Cell   241 TTGGATGCATACAACAGGACATTGACCACTTTGCTCACCCCCCTTGGTGACTCTATCCGTAGGATACAAGAGTCTGTGACTACATCTGGAGGGGGGAGACAGGGGCGCCTTATAGGCGCC 360

Plasmid    361 ATTATTGGCGGTGTGGCTCTTGGGGTTGCAACTGCCGCACAAATAACAGCGGCCGCAGCTCTGATACAAGCCAAACAAAATGCTGCCAACATCCTCCGACTTAAAGAGAGCATTGCCGCA 480
Egg        361 ATTATTGGCGGTGTGGCTCTTGGGGTTGCAACTGCCGCACAAATAACAGCGGCCGCAGCTCTGATACAAGCCAAACAAAATGCTGCCAACATCCTCCGACTTAAAGAGAGCATTGCCGCA 480
1st Cell   361 ATTATTGGCGGTGTGGCTCTTGGGGTTGCAACTGCCGCACAAATAACAGCGGCCGCAGCTCTGATACAAGCCAAACAAAATGCTGCCAACATCCTCCGACTTAAAGAGAGCATTGCCGCA 480
2nd Cell   361 ATTATTGGCGGTGTGGCTCTTGGGGTTGCAACTGCCGCACAAATAACAGCGGCCGCAGCTCTGATACAAGCCAAACAAAATGCTGCCAACATCCTCCGACTTAAAGAGAGCATTGCCGCA 480
3rd Cell   361 ATTATTGGCGGTGTGGCTCTTGGGGTTGCAACTGCCGCACAAATAACAGCGGCCGCAGCTCTGATACAAGCCAAACAAAATGCTGCCAACATCCTCCGACTTAAAGAGAGCATTGCCGCA 480

Plasmid    481 ACCAATGAGGCTGTGCATGAGGTCACTGACGGATTATCGCAACTAGCAGTGGCAGTTGGGAAGATGCAGCAGTTTGTTAATGACCAATTTAATAAAACAGCTCAGGAATTAGACTGCATC 600
Egg        481 ACCAATGAGGCTGTGCATGAGGTCACTGACGGATTATCGCAACTAGCAGTGGCAGTTGGGAAGATGCAGCAGTTTGTTAATGACCAATTTAATAAAACAGCTCAGGAATTAGACTGCATC 600
1st Cell   481 ACCAATGAGGCTGTGCATGAGGTCACTGACGGATTATCGCAACTAGCAGTGGCAGTTGGGAAGATGCAGCAGTTTGTTAATGACCAATTTAATAAAACAGCTCAGGAATTAGACTGCATC 600
2nd Cell   481 ACCAATGAGGCTGTGCATGAGGTCACTGACGGATTATCGCAACTAGCAGTGGCAGTTGGGAAGATGCAGCAGTTTGTTAATGACCAATTTAATAAAACAGCTCAGGAATTAGACTGCATC 600
3rd Cell   481 ACCAATGAGGCTGTGCATGAGGTCACTGACGGATTATCGCAACTAGCAGTGGCAGTTGGGAAGATGCAGCAGTTTGTTAATGACCAATTTAATAAAACAGCTCAGGAATTAGACTGCATC 600

Plasmid    601 AAAATTGCACAGCAAGTTGGTGTAGAGCTCAACCTGTACCTAACCGAATTGACTACAGTATTCGGACCACAAATCACTTCACCTGCCTTAAACAAGCTGACTATTCAGGCACTTTACAAT 720
Egg        601 AAAATTGCACAGCAAGTTGGTGTAGAGCTCAACCTGTACCTAACCGAATTGACTACAGTATTCGGACCACAAATCACTTCACCTGCCTTAAACAAGCTGACTATTCAGGCACTTTACAAT 720
1st Cell   601 AAAATTGCACAGCAAGTTGGTGTAGAGCTCAACCTGTACCTAACCGAATTGACTACAGTATTCGGACCACAAATCACTTCACCTGCCTTAAACAAGCTGACTATTCAGGCACTTTACAAT 720
2nd Cell   601 AAAATTGCACAGCAAGTTGGTGTAGAGCTCAACCTGTACCTAACCGAATTGACTACAGTATTCGGACCACAAATCACTTCACCTGCCTTAAACAAGCTGACTATTCAGGCACTTTACAAT 720
3rd Cell   601 AAAATTGCACAGCAAGTTGGTGTAGAGCTCAACCTGTACCTAACCGAATTGACTACAGTATTCGGACCACAAATCACTTCACCTGCCTTAAACAAGCTGACTATTCAGGCACTTTACAAT 720

Plasmid    721 CTAGCTGGTGGAATATGGATTACTTATTGACTAAGTTAGGTATAGGGAACAATCAACTCAGCTCATTAATCGGTAGCGGCTTAATCACCGGTAACCCTATTCTATACGACTCACAGACT 840
Egg        721 CTAGCTGGTGGAATATGGATTACTTATTGACTAAGTTAGGTATAGGGAACAATCAACTCAGCTCATTAATCGGTAGCGGCTTAATCACCGGTAACCCTATTCTATACGACTCACAGACT 840
1st Cell   721 CTAGCTGGTGGAATATGGATTACTTATTGACTAAGTTAGGTATAGGGAACAATCAACTCAGCTCATTAATCGGTAGCGGCTTAATCACCGGTAACCCTATTCTATACGACTCACAGACT 840
2nd Cell   721 CTAGCTGGTGGAATATGGATTACTTATTGACTAAGTTAGGTATAGGGAACAATCAACTCAGCTCATTAATCGGTAGCGGCTTAATCACCGGTAACCCTATTCTATACGACTCACAGACT 840
3rd Cell   721 CTAGCTGGTGGAATATGGATTACTTATTGACTAAGTTAGGTATAGGGAACAATCAACTCAGCTCATTAATCGGTAGCGGCTTAATCACCGGTAACCCTATTCTATACGACTCACAGACT 840

Plasmid    841 CAACTCTTGGGTATACAGGTAACTCTACCTTCAGTCGGGAACCTAAATAATATGCGTGCCACCTACTTGGAAACCTTATCCGTAAGCACAACCAGGGGATTTGCCTCGGCACTTGTCCCA 960
Egg        841 CAACTCTTGGGTATACAGGTAACTCTACCTTCAGTCGGGAACCTAAATAATATGCGTGCCACCTACTTGGAAACCTTATCCGTAAGCACAACCAGGGGATTTGCCTCGGCACTTGTCCCA 960
1st Cell   841 CAACTCTTGGGTATACAGGTAACTCTACCTTCAGTCGGGAACCTAAATAATATGCGTGCCACCTACTTGGAAACCTTATCCGTAAGCACAACCAGGGGATTTGCCTCGGCACTTGTCCCA 960
2nd Cell   841 CAACTCTTGGGTATACAGGTAACTCTACCTTCAGTCGGGAACCTAAATAATATGCGTGCCACCTACTTGGAAACCTTATCCGTAAGCACAACCAGGGGATTTGCCTCGGCACTTGTCCCA 960
3rd Cell   841 CAACTCTTGGGTATACAGGTAACTCTACCTTCAGTCGGGAACCTAAATAATATGCGTGCCACCTACTTGGAAACCTTATCCGTAAGCACAACCAGGGGATTTGCCTCGGCACTTGTCCCA 960

Plasmid    961 AAAGTGGTGACACAGGTCGGTTCTGTGATAGAAGAACTTGACACCTCATACTGTATAGAAACTGACTTAGATTTATATTGTACAAGAATAGTAACGTTCCCTATGTCCCCTGGTATTTAC 1080
Egg        961 AAAGTGGTGACACAGGTCGGTTCTGTGATAGAAGAACTTGACACCTCATACTGTATAGAAACTGACTTAGATTTATATTGTACAAGAATAGTAACGTTCCCTATGTCCCCTGGTATTTAC 1080
1st Cell   961 AAAGTGGTGACACAGGTCGGTTCTGTGATAGAAGAACTTGACACCTCATACTGTATAGAAACTGACTTAGATTTATATTGTACAAGAATAGTAACGTTCCCTATGTCCCCTGGTATTTAC 1080
2nd Cell   961 AAAGTGGTGACACAGGTCGGTTCTGTGATAGAAGAACTTGACACCTCATACTGTATAGAAACTGACTTAGATTTATATTGTACAAGAATAGTAACGTTCCCTATGTCCCCTGGTATTTAC 1080
3rd Cell   961 AAAGTGGTGACACAGGTCGGTTCTGTGATAGAAGAACTTGACACCTCATACTGTATAGAAACTGACTTAGATTTATATTGTACAAGAATAGTAACGTTCCCTATGTCCCCTGGTATTTAC 1080

Plasmid   1081 TCCTGCTTGAGCGGCAATACATCGGCCTGTATGTACTCAAAGACCGAAGGCGCACTTACTACACCATATATGACTATCAAAGGCTCAGTCATCGCTAACTGCAAGATGACAACATGTAGA 1200
Egg       1081 TCCTGCTTGAGCGGCAATACATCGGCCTGTATGTACTCAAAGACCGAAGGCGCACTTACTACACCATATATGACTATCAAAGGCTCAGTCATCGCTAACTGCAAGATGACAACATGTAGA 1200
1st Cell  1081 TCCTGCTTGAGCGGCAATACATCGGCCTGTATGTACTCAAAGACCGAAGGCGCACTTACTACACCATATATGACTATCAAAGGCTCAGTCATCGCTAACTGCAAGATGACAACATGTAGA 1200
2nd Cell  1081 TCCTGCTTGAGCGGCAATACATCGGCCTGTATGTACTCAAAGACCGAAGGCGCACTTATTACACCATATATGACTATCAAAGGCTCAGTCATCGCTAACTGCAAGATGACAACATGTAGA 1200
3rd Cell  1081 TCCTGCTTGAGCGGCAATACATCGGCCTGTATGTACTCAAAGACCGAAGGCGCACTTACTACACCATATATGACTATCAAAGGCTCAGTCATCGCTAACTGCAAGATGACAACATGTAGA 1200

Plasmid   1201 TGTGTAAACCCCCCGGGTATCATATCGCAAAACTATGGAGAAGCCGTGTCTCTAATAGATAAACAATCATGCAATGTTTTATCCTTAGGCGGGATAACTTTAAGGCTCAGTGGGGAATTC 1320
Egg       1201 TGTGTAAACCCCCCGGGTATCATATCGCAAAACTATGGAGAAGCCGTGTCTCTAATAGATAAACAATCATGCAATGTTTTATCCTTAGGCGGGATAACTTTAAGGCTCAGTGGGGAATTC 1320
1st Cell  1201 TGTGTAAACCCCCCGGGTATCATATCGCAAAACTATGGAGAAGCCGTGTCTCTAATAGATAAACAATCATGCAATGTTTTATCCTTAGGCGGGATAACTTTAAGGCTCAGTGGGGAATTC 1320
2nd Cell  1201 TGTGTAAACCCCCCGGGTATCATATCGCAAAACTATGGAGAAGCCGTGTCTCTAATAGATAAACAATCATGCAATGTTTTATCCTTAGGCGGGATAACTTTAAGGCTCAGTGGGGAATTC 1320
3rd Cell  1201 TGTGTAAACCCCCCGGGTATCATATCGCAAAACTATGGAGAAGCCGTGTCTCTAATAGATAAACAATCATGCAATGTTTTATCCTTAGGCGGGATAACTTTAAGGCTCAGTGGGGAATTC 1320

Plasmid   1321 GATGTAACTTATCAGAAGAATATCTCAATACAAGATTCTCAAGTAATAATAACAGGCAATCTTGATATCTCAACTGAGCTTGGGAATGTCAACAACTCGATCAGTAATGCTTTGAATAAG 1440
Egg       1321 GATGTAACTTATCAGAAGAATATCTCAATACAAGATTCTCAAGTAATAATAACAGGCAATCTTGATATCTCAACTGAGCTTGGGAATGTCAACAACTCGATCAGTAATGCTTTGAATAAG 1440
1st Cell  1321 GATGTAACTTATCAGAAGAATATCTCAATACAAGATTCTCAAGTAATAATAACAGGCAATCTTGATATCTCAACTGAGCTTGGGAATGTCAACAACTCGATCAGTAATGCTTTGAATAAG 1440
2nd Cell  1321 GATGTAACTTATCAGAAGAATATCTCAATACAAGATTCTCAAGTAATAATAACAGGCAATCTTGATATCTCAACTGAGCTTGGGAATGTCAACAACTCGATCAGTAATGCTTTGAATAAG 1440
3rd Cell  1321 GATGTAACTTATCAGAAGAATATCTCAATACAAGATTCTCAAGTAATAATAACAGGCAATCTTGATATCTCAACTGAGCTTGGGAATGTCAACAACTCGATCAGTAATGCTTTGAATAAG 1440

Plasmid   1441 TTAGAGGAAAGCAACAGAAAACTAGACAAAGTCAATGTCAAACTGACCAGCACATCTGCTCTCATTACCTATATCGTTTTGACTATCATATCTCTTGTTTTTGGTATACTTAGCCTGATT 1560
Egg       1441 TTAGAGGAAAGCAACAGAAAACTAGACAAAGTCAATGTCAAACTGACCAGCACATCTGCTCTCATTACCTATATCGTTTTGACTATCATATCTCTTGTTTTTGGTATACTTAGCCTGATT 1560
1st Cell  1441 TTAGAGGAAAGCAACAGAAAACTAGACAAAGTCAATGTCAAACTGACCAGCACATCTGCTCTCATTACCTATATCGTTTTGACTATCATATCTCTTGTTTTTGGTATACTTAGCCTGATT 1560
2nd Cell  1441 TTAGAGGAAAGCAACAGAAAACTAGACAAAGTCAATGTCAAACTGACCAGCACATCTGCTCTCATTACCTATATCGTTTTGACTATCATATCTCTTGTTTTTGGTATACTTAGCCTGATT 1560
3rd Cell  1441 TTAGAGGAAAGCAACAGAAAACTAGACAAAGTCAATGTCAAACTGACCAGCACATCTGCTCTCATTACCTATATCGTTTTGACTATCATATCTCTTGTTTTTGGTATACTTAGCCTGATT 1560

Plasmid   1561 CTAGCATGCTACCTAATGTACAAGCAAAAGGCGCAACAAAAGACCTTATTATGGCTTGGGAATAATACCCTAGATCAGATGAGAGCCACTACAAAAATGTGA 1662
Egg       1561 CTAGCATGCTACCTAATGTACAAGCAAAAGGCGCAACAAAAGACCTTATTATGGCTTGGGAATAATACCCTAGATCAGATGAGAGCCACTACAAAAATGTGA 1662
1st Cell  1561 CTAGCATGCTACCTAATGTACAAGCAAAAGGCGCAACAAAAGACCTTATTATGGCTTGGGAATAATACCCTAGATCAGATGAGAGCCACTACAAAAATGTGA 1662
2nd Cell  1561 CTAGCATGCTACCTAATGTACAAGCAAAAGGCGCAACAAAAGACCTTATTATGGCTTGGGAATAATACCCTAGATCAGATGAGAGCCACTACAAAAATGTGA 1662
3rd Cell  1561 CTAGCATGCTACCTAATGTACAAGCAAAAGGCGCAACAAAAGACCTTATTATGGCTTGGGAATAATACCCTAGATCAGATGAGAGCCACTACAAAAATGTGA 1662
```

Plasmid : Plasmids for rescue experiments
Egg : Viruses were propagated in embryonated eggs
1st Cell :
2nd Cell :
3rd Cell :
} Each passage viruses in HEp-2 cells

rNDV-2HN (APMV-2 HN gene)

```
Plasmid     1 ATGGATTTCCCATCTAGGGAGAACCTGGCAGCAGGTGACATATCGGGGCGGAAGACTTGGAGATTACTGTTCCGGATCCTCACATTGAGCATAGGTGTGGTCTGTCTTGCCATCAATATT 120
Egg         1 ATGGATTTCCCATCTAGGGAGAACCTGGCAGCAGGTGACATATCGGGGCGGAAGACTTGGAGATTACTGTTCCGGATCCTCACATTGAGCATAGGTGTGGTCTGTCTTGCCATCAATATT 120
1st Cell    1 ATGGATTTCCCATCTAGGGAGAACCTGGCAGCAGGTGACATATCGGGGCGGAAGACTTGGAGATTACTGTTCCGGATCCTCACATTGAGCATAGGTGTGGTCTGTCTTGCCATCAATATT 120
2nd Cell    1 ATGGATTTCCCATCTAGGGAGAACCTGGCAGCAGGTGACATATCGGGGCGGAAGACTTGGAGATTACTGTTCCGGATCCTCACATTGAGCATAGGTGTGGTCTGTCTTGCCATCAATATT 120
3rd Cell    1 ATGGATTTCCCATCTAGGGAGAACCTGGCAGCAGGTGACATATCGGGGCGGAAGACTTGGAGATTACTGTTCCGGATCCTCACATTGAGCATAGGTGTGGTCTGTCTTGCCATCAATATT 120

Plasmid   121 GCCACAATTGCAAAATTGGATCACCTGGATAACATGGCTTCGAACACATGGACAACAACTGAGGCTGACCGTGTGATATCTAGCATCACGACTCCGCTCAAAGTCCCTGTCAACCAGATT 240
Egg       121 GCCACAATTGCAAAATTGGATCACCTGGATAACATGGCTTCGAACACATGGACAACAACTGAGGCTGACCGTGTGATATCTAGCATCACGACTCCGCTCAAAGTCCCTGTCAACCAGATT 240
1st Cell  121 GCCACAATTGCAAAATTGGATCACCTGGATAACATGGCTTCGAACACATGGACAACAACTGAGGCTGACCGTGTGATATCTAGCATCACGACTCCGCTCAAAGTCCCTGTCAACCAGATT 240
2nd Cell  121 GCCACAATTGCAAAATTGGATCACCTGGATAACATGGCTTCGAACACATGGACAACAACTGAGGCTGACCGTGTGATATCTAGCATCACGACTCCGCTCAAAGTCCCTGTCAACCAGATT 240
3rd Cell  121 GCCACAATTGCAAAATTGGATCACCTGGATAACATGGCTTCGAACACATGGACAACAACTGAGGCTGACCGTGTGATATCTAGCATCACGACTCCGCTCAAAGTCCCTGTCAACCAGATT 240

Plasmid   241 AATGACATGTTTCGGATTGTAGCGCTTGACCTACCTCTGCAGATGACATCATTACAGAAAGAAACAGCATCCCAAGTCGGGTTCTTGGCTGAAAGTATCAACAATGTTTTATCCAAGAAT 360
Egg       241 AATGACATGTTTCGGATTGTAGCGCTTGACCTACCTCTGCAGATGACATCATTACAGAAAGAAACAGCATCCCAAGTCGGGTTCTTGGCTGAAAGTATCAACAATGTTTTATCCAAGAAT 360
1st Cell  241 AATGACATGTTTCGGATTGTAGCGCTTGACCTACCTCTGCAGATGACATCATTACAGAAAGAAACAGCATCCCAAGTCGGGTTCTTGGCTGAAAGTATCAACAATGTTTTATCCAAGAAT 360
2nd Cell  241 AATGACATGTTTCGGATTGTAGCGCTTGACCTACCTCTGCAGATGACATCATTACAGAAAGAAACAGCATCCCAAGTCGGGTTCTTGGCTGAAAGTATCAACAATGTTTTATCCAAGAAT 360
3rd Cell  241 AATGACATGTTTCGGATTGTAGCGCTTGACCTACCTCTGCAGATGACATCATTACAGAAAGAAACAGCATCCCAAGTCGGGTTCTTGGCTGAAAGTATCAACAATGTTTTATCCAAGAAT 360

Plasmid   361 GGATCTGCAGGCCTGGTTCTTGTTAATGACCCTGAATATGCAGGGGGGATCGCTGTCAGCTTGTACCAAGGAGATGCATCTGCAGGCCTAAATTTCCAGCCCATTTCTTTAATAGAACAT 480
Egg       361 GGATCTGCAGGCCTGGTTCTTGTTAATGACCCTGAATATGCAGGGGGGATCGCTGTCAGCTTGTACCAAGGAGATGCATCTGCAGGCCTAAATTTCCAGCCCATTTCTTTAATAGAACAT 480
1st Cell  361 GGATCTGCAGGCCTGGTTCTTGTTAATGACCCTGAATATGCAGGGGGGATCGCTGTCAGCTTGTACCAAGGAGATGCATCTGCAGGCCTAAATTTCCAGCCCATTTCTTTAATAGAACAT 480
2nd Cell  361 GGATCTGCAGGCCTGGTTCTTGTTAATGACCCTGAATATGCAGGGGGGATCGCTGTCAGCTTGTACCAAGGAGATGCATCTGCAGGCCTAAATTTCCAGCCCATTTCTTTAATAGAACAT 480
3rd Cell  361 GGATCTGCAGGCCTGGTTCTTGTTAATGACCCTGAATATGCAGGGGGGATCGCTGTCAGCTTGTACCAAGGAGATGCATCTGCAGGCCTAAATTTCCAGCCCATTTCTTTAATAGAACAT 480

Plasmid   481 CCAAGTTTTGTCCCTGGTCCTACTACTGCTAAGGGCTGTATAAGGATCCCGACCTTCCATATGGGCCCTTCACATTGGTGTTACTCACATAACATCATTGCATCAGGTTGCCAGGATGCG 600
Egg       481 CCAAGTTTTGTCCCTGGTCCTACTACTGCTAAGGGCTGTATAAGGATCCCGACCTTCCATATGGGCCCTTCACATTGGTGTTACTCACATAACATCATTGCATCAGGTTGCCAGGATGCG 600
1st Cell  481 CCAAGTTTTGTCCCTGGTCCTACTACTGCTAAGGGCTGTATAAGGATCCCGACCTTCCATATGGGCCCTTCACATTGGTGTTACTCACATAACATCATTGCATCAGGTTGCCAGGATGCG 600
2nd Cell  481 CCAAGTTTTGTCCCTGGTCCTACTACTGCTAAGGGCTGTATAAGGATCCCGACCTTCCATATGGGCCCTTCACATTGGTGTTACTCACATAACATCATTGCATCAGGTTGCCAGGATGCG 600
3rd Cell  481 CCAAGTTTTGTCCCTGGTCCTACTACTGCTAAGGGCTGTATAAGGATCCCGACCTTCCATATGGGCCCTTCACATTGGTGTTACTCACATAACATCATTGCATCAGGTTGCCAGGATGCG 600

Plasmid   601 AGCCACTCCAGTATGTATATCTCTCTGGGGGTGCTGAAAGCATCGCAGACCGGGTCGCCTATCTTCTTGACAACGGCCAGCCAGCTCGTGGATGACAACATCAACCGGAAGTCATGCAGC 720
Egg       601 AGCCACTCCAGTATGTATATCTCTCTGGGGGTGCTGAAAGCATCGCAGACCGGGTCGCCTATCTTCTTGACAACGGCCAGCCAGCTCGTGGATGACAACATCAACCGGAAGTCATGCAGC 720
1st Cell  601 AGCCACTCCAGTATGTATATCTCTCTGGGGGTGCTGAAAGCATCGCAGACCGGGTCGCCTATCTTCTTGACAACGGCCAGCCAGCTCGTGGATGACAACATCAACCGGAAGTCATGCAGC 720
2nd Cell  601 AGCCACTCCAGTATGTATATCTCTCTGGGGGTGCTGAAAGCATCGCAGACCGGGTCGCCTATCTTCTTGACAACGGCCAGCCAGCTCGTGGATGACAACATCAACCGGAAGTCATGCAGC 720
3rd Cell  601 AGCCACTCCAGTATGTATATCTCTCTGGGGGTGCTGAAAGCATCGCAGACCGGGTCGCCTATCTTCTTGACAACGGCCAGCCAGCTCGTGGATGACAACATCAACCGGAAGTCATGCAGC 720

Plasmid   721 ATCGTAGCCTCAAAATACGGTTGTGATATCCTATGCAGTATTGTGATTGAAACAGAGGATGAGGATTATAGGTCTGATCCGGCTACTAGCATGATTATAGGTAGGCTGTTCTTCAACGGG 840
Egg       721 ATCGTAGCCTCAAAATACGGTTGTGATATCCTATGCAGTATTGTGATTGAAACAGAGGATGAGGATTATAGGTCTGATCCGGCTACTAGCATGATTATAGGTAGGCTGTTCTTCAACGGG 840
1st Cell  721 ATCGTAGCCTCAAAATACGGTTGTGATATCCTATGCAGTATTGTGATTGAAACAGAGGATGAGGATTATAGGTCTGATCCGGCTACTAGCATGATTATAGGTAGGCTGTTCTTCAACGGG 840
2nd Cell  721 ATCGTAGCCTCAAAATACGGTTGTGATATCCTATGCAGTATTGTGATTGAAACAGAGGATGAGGATTATAGGTCTGATCCGGCTACTAGCATGATTATAGGTAGGCTGTTCTTCAACGGG 840
3rd Cell  721 ATCGTAGCCTCAAAATACGGTTGTGATATCCTATGCAGTATTGTGATTGAAACAGAGGATGAGGATTATAGGTCTGATCCGGCTACTAGCATGATTATAGGTAGGCTGTTCTTCAACGGG 840

Plasmid   841 TCATACACAGAGAGCAAGATTAACACAGGGTCCATCTTCAGTCTATTCTCTGCTAACTACCCTGCGGTGGGGTCGGGTATTGTAGTCGGGGATGAAGCCGCATTCCCAATATATGGTGGG 960
Egg       841 TCATACACAGAGAGCAAGATTAACACAGGGTCCATCTTCAGTCTATTCTCTGCTAACTACCCTGCGGTGGGGTCGGGTATTGTAGTCGGGGATGAAGCCGCATTCCCAATATATGGTGGG 960
1st Cell  841 TCATACACAGAGAGCAAGATTAACACAGGGTCCATCTTCAGTCTATTCTCTGCTAACTACCCTGCGGTGGGGTCGGGTATTGTAGTCGGGGATGAAGCCGCATTCCCAATATATGGTGGG 960
2nd Cell  841 TCATACACAGAGAGCAAGATTAACACAGGGTCCATCTTCAGTCTATTCTCTGCTAACTACCCTGCGGTGGGGTCGGGTATTGTAGTCGGGGATGAAGCCGCATTCCCAATATATGGTGGG 960
3rd Cell  841 TCATACACAGAGAGCAAGATTAACACAGGGTCCATCTTCAGTCTATTCTCTGCTAACTACCCTGCGGTGGGGTCGGGTATTGTAGTCGGGGATGAAGCCGCATTCCCAATATATGGTGGG 960

Plasmid   961 GTCAAGCAGAACACATGGTTGTTCAACCAGCTCAAGGATTTTGGTTACTTCACCCATAATGATGTGTACAAGTGCAATCGGACTGATATACAGCAAACTATCCTGGATGCATACAGGCCA 1080
Egg       961 GTCAAGCAGAACACATGGTTGTTCAACCAGCTCAAGGATTTTGGTTACTTCACCCATAATGATGTGTACAAGTGCAATCGGACTGATATACAGCAAACTATCCTGGATGCATACAGGCCA 1080
1st Cell  961 GTCAAGCAGAACACATGGTTGTTCAACCAGCTCAAGGATTTTGGTTACTTCACCCATAATGATGTGTACAAGTGCAATCGGACTGATATACAGCAAACTATCCTGGATGCATACAGGCCA 1080
2nd Cell  961 GTCAAGCAGAACACATGGTTGTTCAACCAGCTCAAGGATTTTGGTTACTTCACCCATAATGATGTGTACAAGTGCAATCGGACTGATATACAGCAAACTATCCTGGATGCATACAGGCCA 1080
3rd Cell  961 GTCAAGCAGAACACATGGTTGTTCAACCAGCTCAAGGATTTTGGTTACTTCACCCATAATGATGTGTACAAGTGCAATCGGACTGATATACAGCAAACTATCCTGGGTGCATACAGGCCA 1080

Plasmid  1081 CCTAAAATCTCAGGAAGGTTATGGGTACAAGGCATCCTATTGTGCCCAGTTTCACTGAGACCTGATCCTGGCTGTCGCTTAAAGGTGTTCAATACCAGCAATGTGATGATGGGGGCAGAA 1200
Egg      1081 CCTAAAATCTCAGGAAGGTTATGGGTACAAGGCATCCTATTGTGCCCAGTTTCACTGAGACCTGATCCTGGCTGTCGCTTAAAGGTGTTCAATACCAGCAATGTGATGATGGGGGCAGAA 1200
1st Cell 1081 CCTAAAATCTCAGGAAGGTTATGGGTACAAGGCATCCTATTGTGCCCAGTTTCACTGAGACCTGATCCTGGCTGTCGCTTAAAGGTGTTCAATACCAGCAATGTGATGATGGGGGCAGAA 1200
2nd Cell 1081 CCTAAAATCTCAGGAAGGTTATGGGTACAAGGCATCCTATTGTGCCCAGTTTCACTGAGACCTGATCCTGGCTGTCGCTTAAAGGTGTTCAATACCAGCAATGTGATGATGGGGGCAGAA 1200
3rd Cell 1081 CCTAAAATCTCAGGAAGGTTATGGGTACAAGGCATCCTATTGTGCCCAGTTTCACTGAGACCTGATCCTGGCTGTCGCTTAAAGGTGTTCAATACCAGCAATGTGATGATGGGGGCAGAA 1200

Plasmid  1201 GCGAGGTTGATCCAAGTAGGCTCAACCGTGTATCTATACCAACGCTCATCCTCATGGTGGGTGGTAGGACTGACTTACAAATTAGATGTGTCAGAAATAACTTCACAGACAGGTAACACA 1320
Egg      1201 GCGAGGTTGATCCAAGTAGGCTCAACCGTGTATCTATACCAACGCTCATCCTCATGGTGGGTGGTAGGACTGACTTACAAATTAGATGTGTCAGAAATAACTTCACAGACAGGTAACACA 1320
1st Cell 1201 GCGAGGTTGATCCAAGTAGGCTCAACCGTGTATCTATACCAACGCTCATCCTCATGGTGGGTGGTAGGACTGACTTACAAATTAGATGTGTCAGAAATAACTTCACAGACAGGTAACACA 1320
2nd Cell 1201 GCGAGGTTGATCCAAGTAGGCTCAACCGTGTATCTATACCAACGCTCATCCTCATGGTGGGTGGTAGGACTGACTTACAAATTAGATGTGTCAGAAATAACTTCACAGACAGGTAACACA 1320
3rd Cell 1201 GCGAGGTTGATCCAAGTAGGCTCAACCGTGTATCTATACCAACGCTCATCCTCATGGTGGGTGGTAGGACTGACTTACAAATTAGATGTGTCAGAAATAACTTCACAGACAGGTAACACA 1320

Plasmid  1321 CTCAACCATGTAGACCCCATTGCCCATACAAAGTTCCCAAGACCATCTTTCGGGCGAGATGCGTGTGCGAGGCCAAACATATGCCCTGCTGTCTGTGTCTCCGGAGTTTATCAGGACATT 1440
Egg      1321 CTCAACCATGTAGACCCCATTGCCCATACAAAGTTCCCAAGACCATCTTTCGGGCGAGATGCGTGTGCGAGGCCAAACATATGCCCTGCTGTCTGTGTCTCCGGAGTTTATCAGGACATT 1440
1st Cell 1321 CTCAACCATGTAGACCCCATTGCCCATACAAAGTTCCCAAGACCATCTTTCGGGCGAGATGCGTGTGCGAGGCCAAACATATGCCCTGCTGTCTGTGTCTCCGGAGTTTATCAGGACATT 1440
2nd Cell 1321 CTCAACCATGTAGACCCCATTGCCCATACAAAGTTCCCAAGACCATCTTTCGGGCGAGATGCGTGTGCGAGGCCAAACATATGCCCTGCTGTCTGTGTCTCCGGAGTTTATCAGGACATT 1440
3rd Cell 1321 CTCAACCATGTAGACCCCATTGCCCATACAAAGTTCCCAAGACCATCTTTCGGGCGAGATGCGTGTGCGAGGCCAAACATATGCCCTGCTGTCTGTGTCTCCGGAGTTTATCAGGACATT 1440

Plasmid  1441 TGGCCGATCAGTACAGCCACCAATAACAGCAACATTGTGTGGGTTGGACAGTACTTAGAAGCATTCTATTCCAGGAAATACCCAAGAATAGGGATAGCAACCCAGTATGAGTGGAAAGTC 1560
Egg      1441 TGGCCGATCAGTACAGCCACCAATAACAGCAACATTGTGTGGGTTGGACAGTACTTAGAAGCATTCTATTCCAGGAAATACCCAAGAATAGGGATAGCAACCCAGTATGAGTGGAAAGTC 1560
1st Cell 1441 TGGCCGATCAGTACAGCCACCAATAACAGCAACATTGTGTGGGTTGGACAGTACTTAGAAGCATTCTATTCCAGGAAATACCCAAGAATAGGGATAGCAACCCAGTATGAGTGGAAAGTC 1560
2nd Cell 1441 TGGCCGATCAGTACAGCCACCAATAACAGCAACATTGTGTGGGTTGGACAGTACTTAGAAGCATTCTATTCCAGGAAATACCCAAGAATAGGGATAGCAACCCAGTATGAGTGGAAAGTC 1560
3rd Cell 1441 TGGCCGATCAGTACAGCCACCAATAACAGCAACATTGTGTGGGTTGGACAGTACTTAGAAGCATTCTATTCCAGGAAATACCCAAGAATAGGGATAGCAACCCAGTATGAGTGGAAAGTC 1560

Plasmid  1561 ACCAACCAGCTGTTCAATTCGAATACTGAGGGAGGGTACTCAACCACAACATGCTTCCGGAACACCAAACGGGACAAGGCATATTGTGTAGTGATATCAGAGTACGCTGATGGGGTGTTC 1680
Egg      1561 ACCAACCAGCTGTTCAATTCGAATACTGAGGGAGGGTACTCAACCACAACATGCTTCCGGAACACCAAACGGGACAAGGCATATTGTGTAGTGATATCAGAGTACGCTGATGGGGTGTTC 1680
1st Cell 1561 ACCAACCAGCTGTTCAATTCGAATACTGAGGGAGGGTACTCAACCACAACATGCTTCCGGAACACCAAACGGGACAAGGCATATTGTGTAGTGATATCAGAGTACGCTGATGGGGTGTTC 1680
2nd Cell 1561 ACCAACCAGCTGTTCAATTCGAATACTGAGGGAGGGTACTCAACCACAACATGCTTCCGGAACACCAAACGGGACAAGGCATATTGTGTAGTGATATCAGAGTACGCTGATGGGGTGTTC 1680
3rd Cell 1561 ACCAACCAGCTGTTCAATTCGAATACTGAGGGAGGGTACTCAACCACAACATGCTTCCGGAACACCAAACGGGACAAGGCATATTGTGTAGTGATATCAGAGTACGCTGATGGGGTGTTC 1680

Plasmid  1681 GGATCATACAGGATCGTTCCTCAGCTTATAGAGATTAGAACGACCACCGGTAAATCTGAGTGA 1743
Egg      1681 GGATCATACAGGATCGTTCCTCAGCTTATAGAGATTAGAACGACCACCGGTAAATCTGAGTGA 1743
1st Cell 1681 GGATCATACAGGATCGTTCCTCAGCTTATAGAGATTAGAACGACCACCGGTAAATCTGAGTGA 1743
2nd Cell 1681 GGATCATACAGGATCGTTCCTCAGCTTATAGAGATTAGAACGACCACCGGTAAATCTGAGTGA 1743
3rd Cell 1681 GGATCATACAGGATCGTTCCTCAGCTTATAGAGATTAGAACGACCACCGGTAAATCTGAGTGA 1743
```

Plasmid : Plasmids for rescue experiments
Egg : Viruses were propagated in embryonated eggs
1st Cell :
2nd Cell : Each passage viruses in HEp-2 cells
3rd Cell :

rNDV-2F/2HN (APMV-2 F gene)

```
Plasmid     1 ATGAATCAAGCACTCGTGATTTTGTTGGTATCTTTCCAGCTCGGCGTTGCCTTAGATAACTCAGTGTTGGCTCCAATAGGAGTAGCTAGCGCACAGGAGTGGCAACTGGCGGCATATACA 120
Egg         1 ATGAATCAAGCACTCGTGATTTTGTTGGTATCTTTCCAGCTCGGCGTTGCCTTAGATAACTCAGTGTTGGCTCCAATAGGAGTAGCTAGCGCACAGGAGTGGCAACTGGCGGCATATACA 120
1st Cell    1 ATGAATCAAGCACTCGTGATTTTGTTGGTATCTTTCCAGCTCGGCGTTGCCTTAGATAACTCAGTGTTGGCTCCAATAGGAGTAGCTAGCGCACAGGAGTGGCAACTGGCGGCATATACA 120
2nd Cell    1 ATGAATCAAGCACTCGTGATTTTGTTGGTATCTTTCCAGCTCGGCGTTGCCTTAGATAACTCAGTGTTGGCTCCAATAGGAGTAGCTAGCGCACAGGAGTGGCAACTGGCGGCATATACA 120
3rd Cell    1 ATGAATCAAGCACTCGTGATTTTGTTGGTATCTTTCCAGCTCGGCGTTGCCTTAGATAACTCAGTGTTGGCTCCAATAGGAGTAGCTAGCGCACAGGAGTGGCAACTGGCGGCATATACA 120

Plasmid   121 ACGACCCTCACAGGGACCATCGCAGTGAGATTTATCCCGGTCCTGCCTGGGAACCTATCAACATGTGCACAGGAGACGCTGCAGGAATATAATAGAACTGTGACTAATATCTTAGGCCCG 240
Egg       121 ACGACCCTCACAGGGACCATCGCAGTGAGATTTATCCCGGTCCTGCCTGGGAACCTATCAACATGTGCACAGGAGACGCTGCAGGAATATAATAGAACTGTGACTAATATCTTAGGCCCG 240
1st Cell  121 ACGACCCTCACAGGGACCATCGCAGTGAGATTTATCCCGGTCCTGCCTGGGAACCTATCAACATGTGCACAGGAGACGCTGCAGGAATATAATAGAACTGTGACTAATATCTTAGGCCCG 240
2nd Cell  121 ACGACCCTCACAGGGACCATCGCAGTGAGATTTATCCCGGTCCTGCCTGGGAACCTATCAACATGTGCACAGGAGACGCTGCAGGAATATAATAGAACTGTGACTAATATCTTAGGCCCG 240
3rd Cell  121 ACGACCCTCACAGGGACCATCGCAGTGAGATTTATCCCGGTCCTGCCTGGGAACCTATCAACATGTGCACAGGAGACGCTGCAGGAATATAATAGAACTGTGACTAATATCTTAGGCCCG 240

Plasmid   241 TTGAGAGAGAACTTGGATGCTCTCCTATCTGACTTCGATAAACCTGCATCGAGGTTCGTGGGCGCCATCATTGGGTCGGTGGCCTTGGGGGTAGCAACAGCTGCACAAATCACAGCCGCC 360
Egg       241 TTGAGAGAGAACTTGGATGCTCTCCTATCTGACTTCGATAAACCTGCATCGAGGTTCGTGGGCGCCCTCATTGGGTCGGTGGCCTTGGGGGTAGCAACAGCTGCACAAATCACAGCCGCC 360
1st Cell  241 TTGAGAGAGAACTTGGATGCTCTCCTATCTGACTTCGATAAACCTGCATCGAGGTTCGTGGGCGCCCTCATTGGGTCGGTGGCCTTGGGGGTAGCAACAGCTGCACAAATCACAGCCGCC 360
2nd Cell  241 TTGAGAGAGAACTTGGATGCTCTCCTATCTGACTTCGATAAACCTGCATCGAGGTTCGTGGGCGCCCTCATTGGGTCGGTGGCCTTGGGGGTAGCAACAGCTGCACAAATCACAGCCGCC 360
3rd Cell  241 TTGAGAGAGAACTTGGATGCTCTCCTATCTGACTTCGATAAACCTGCATCGAGGTTCGTGGGCGCCCTCATTGGGTCGGTGGCCTTGGGGGTAGCAACAGCTGCACAAATCACAGCCGCC 360

Plasmid   361 GTGGCTCTCAATCAAGCACAAGAGAATGCCCGGAATATATGGCGTCTCAAGGAATCGATAAAGAAAACCAATGAGGCTGTGTTGGAATTGAAGGAAGGACTTGCAACGACTGCTATAGCT 480
Egg       361 GTGGCTCTCAATCAAGCACAAGAGAATGCCCGGAATATATGGCGTCTCAAGGAATCGATAAAGAAAACCAATGAGGCTGTGTTGGAATTGAAGGAAGGACTTGCAACGACTGCTATAGCT 480
1st Cell  361 GTGGCTCTCAATCAAGCACAAGAGAATGCCCGGAATATATGGCGTCTCAAGGAATCGATAAAGAAAACCAATGAGGCTGTGTTGGAATTGAAGGAAGGACTTGCAACGACTGCTATAGCT 480
2nd Cell  361 GTGGCTCTCAATCAAGCACAAGAGAATGCCCGGAATATATGGCGTCTCAAGGAATCGATAAAGAAAACCAATGAGGCTGTGTTGGAATTGAAGGAAGGACTTGCAACGACTGCTATAGCT 480
3rd Cell  361 GTGGCTCTCAATCAAGCACAAGAGAATGCCCGGAATATATGGCGTCTCAAGGAATCGATAAAGAAAACCAATGAGGCTGTGTTGGAATTGAAGGAAGGACTTGCAACGACTGCTATAGCT 480

Plasmid   481 TTGGACAAAGTGCAAAAGTTTATCAATGATGATATTATACCACAGATTAAGGACATTGACTGCCAGGTAGTTGCAAATAAATTAGGCGTCTACCTCTCCTTATACTTAACAGAGCTTACA 600
Egg       481 TTGGACAAAGTGCAAAAGTTTATCAATGATGATATTATACCACAGATTAAGGACATTGACTGCCAGGTAGTTGCAAATAAATTAGGCGTCTACCTCTCCTTATACTTAACAGAGCTTACA 600
1st Cell  481 TTGGACAAAGTGCAAAAGTTTATCAATGATGATATTATACCACAGATTAAGGACATTGACTGCCAGGTAGTTGCAAATAAATTAGGCGTCTACCTCTCCTTATACTTAACAGAGCTTACA 600
2nd Cell  481 TTGGACAAAGTGCAAAAGTTTATCAATGATGATATTATACCACAGATTAAGGACATTGACTGCCAGGTAGTTGCAAATAAATTAGGCGTCTACCTCTCCTTATACTTAACAGAGCTTACA 600
3rd Cell  481 TTGGACAAAGTGCAAAAGTTTATCAATGATGATATTATACCACAGATTAAGGACATTGACTGCCAGGTAGTTGCAAATAAATTAGGCGTCTACCTCTCCTTATACTTAACAGAGCTTACA 600

Plasmid   601 ACTGTATTTGGTTCTCAGATCACTAATCCTGCATTATCAACGCTCTCTTACCAGGCGCTGTACAGCTTATGTGGAGGGGATATGGGAAAGCTAACTGAGCTGATCGGTGTCAATGCAAAG 720
Egg       601 ACTGTATTTGGTTCTCAGATCACTAATCCTGCATTATCAACGCTCTCTTACCAGGCGCTGTACAGCTTATGTGGAGGGGATATGGGAAAGCTAACTGAGCTGATCGGTGTCAATGCAAAG 720
1st Cell  601 ACTGTATTTGGTTCTCAGATCACTAATCCTGCATTATCAACGCTCTCTTACCAGGCGCTGTACAGCTTATGTGGAGGGGATATGGGAAAGCTAACTGAGCTGATCGGTGTCAATGCAAAG 720
2nd Cell  601 ACTGTATTTGGTTCTCAGATCACTAATCCTGCATTATCAACGCTCTCTTACCAGGCGCTGTACAGCTTATGTGGAGGGGATATGGGAAAGCTAACTGAGCTGATCGGTGTCAATGCAAAG 720
3rd Cell  601 ACTGTATTTGGTTCTCAGATCACTAATCCTGCATTATCAACGCTCTCTTACCAGGCGCTGTACAGCTTATGTGGAGGGGATATGGGAAAGCTAACTGAGCTGATCGGTGTCAATGCAAAG 720

Plasmid   721 GATGTGGGATCCCTCTACGAGGCTAACCTCATAACCGGCCAAATCGTTGGATATGACCCTGAACTACAGATAATCCTCATACAAGTATCTTACCCAAGTGTGTCTGAAGTGACAGGAGTC 840
Egg       721 GATGTGGGATCCCTCTACGAGGCTAACCTCATAACCGGCCAAATCGTTGGATATGACCCTGAACTACAGATAATCCTCATACAAGTATCTTACCCAAGTGTGTCTGAAGTGACAGGAGTC 840
1st Cell  721 GATGTGGGATCCCTCTACGAGGCTAACCTCATAACCGGCCAAATCGTTGGATATGACCCTGAACTACAGATAATCCTCATACAAGTATCTTACCCAAGTGTGTCTGAAGTGACAGGAGTC 840
2nd Cell  721 GATGTGGGATCCCTCTACGAGGCTAACCTCATAACCGGCCAAATCGTTGGATATGACCCTGAACTACAGATAATCCTCATACAAGTATCTTACCCAAGTGTGTCTGAAGTGACAGGAGTC 840
3rd Cell  721 GATGTGGGATCCCTCTACGAGGCTAACCTCATAACCGGCCAAATCGTTGGATATGACCCTGAACTACAGATAATCCTCATACAAGTATCTTACCCAAGTGTGTCTGAAGTGACAGGAGTC 840

Plasmid   841 CGGGCTACTGAGTTAGTCACTGTCAGTGTCACTACACCAAAAGGAGAAGGGCAGGCAATTGTTCCGAGATATGTGGCACAGAGTAGAGTGCTGACAGAGGAGTTGGATGTCTCGACTTGT 960
Egg       841 CGGGCTACTGAGTTAGTCACTGTCAGTGTCACTACACCAAAAGGAGAAGGGCAGGCAATTGTTCCGAGATATGTGGCACAGAGTAGAGTGCTGACAGAGGAGTTGGATGTCTCGACTTGT 960
1st Cell  841 CGGGCTACTGAGTTAGTCACTGTCAGTGTCACTACACCAAAAGGAGAAGGGCAGGCAATTGTTCCGAGATATGTGGCACAGAGTAGAGTGCTGACAGAGGAGTTGGATGTCTCGACTTGT 960
2nd Cell  841 CGGGCTACTGAGTTAGTCACTGTCAGTGTCACTACACCAAAAGGAGAAGGGCAGGCAATTGTTCCGAGATATGTGGCACAGAGTAGAGTGCTGACAGAGGAGTTGGATGTCTCGACTTGT 960
3rd Cell  841 CGGGCTACTGAGTTAGTCACTGTCAGTGTCACTACACCAAAAGGAGAAGGGCAGGCAATTGTTCCGAGATATGTGGCACAGAGTAGAGTGCTGACAGAGGAGTTGGATGTCTCGACTTGT 960

Plasmid   961 AGGTTTAGCAAAACAACTCTTTATTGTAGGTCGATTCTCACACGGCCCCTACCAACTTTGATCGCCAGCTGCCTGTCAGGGAAGTACGACGATTGTCAGTACACAACAGAGATAGGAGCG 1080
Egg       961 AGGTTTAGCAAAACAACTCTTTATTGTAGGTCGATTCTCACACGGCCCCTACCAACTTTGATCGCCAGCTGCCTGTCAGGGAAGTACGACGATTGTCAGTACACAACAGAGATAGGAGCG 1080
1st Cell  961 AGGTTTAGCAAAACAACTCTTTATTGTAGGTCGATTCTCACACGGCCCCTACCAACTTTGATCGCCAGCTGCCTGTCAGGGAAGTACGACGATTGTCAGTACACAACAGAGATAGGAGCG 1080
2nd Cell  961 AGGTTTAGCAAAACAACTCTTTATTGTAGGTCGATTCTCACACGGCCCCTACCAACTTTGATCGCCAGCTGCCTGTCAGGGAAGTACGACGATTGTCAGTACACAACAGAGATAGGAGCG 1080
3rd Cell  961 AGGTTTAGCAAAACAACTCTTTATTGTAGGTCGATTCTCACACGGCCCCTACCAACTTTGATCGCCAGCTGCCTGTCAGGGAAGTACGACGATTGTCAGTACACAACAGAGATAGGAGCG 1080

Plasmid  1081 CTATCTTCGAGATTCATCACAGTCAATGGTGGAGTCCTTGCAAACTGCAGAGCAATTGTGTGTAAGTGTGTCTCACCCCCGCATATAATACCACAAAACGACATTGGCTCCGTAACAGTT 1200
Egg      1081 CTATCTTCGAGATTCATCACAGTCAATGGTGGAGTCCTTGCAAACTGCAGAGCAATTGTGTGTAAGTGTGTCTCACCCCCGCATATAATACCACAAAACGACATTGGCTCCGTAACAGTT 1200
1st Cell 1081 CTATCTTCGAGATTCATCACAGTCAATGGTGGAGTCCTTGCAAACTGCAGAGCAATTGTGTGTAAGTGTGTCTCACCCCCGCATATAATACCACAAAACGACATTGGCTCCGTAACAGTT 1200
2nd Cell 1081 CTATCTTCGAGATTCATCACAGTCAATGGTGGAGTCCTTGCAAACTGCAGAGCAATTGTGTGTAAGTGTGTCTCACCCCCGCATATAATACCACAAAACGACATTGGCTCCGTAACAGTT 1200
3rd Cell 1081 CTATCTTCGAGATTCATCACAGTCAATGGTGGAGTCCTTGCAAACTGCAGAGCAATTGTGTGTAAGTGTGTCTCACCCCCGCATATAATACCACAAAACGACATTGGCTCCGTAACAGTT 1200

Plasmid  1201 ATTGACTCAAGTATATGCAAGGAAGTTGTCTTAGAGAGTGTGCAGCTTAGGTTAGAAGGAAAGCTGTCATCCCAATACTTCTCCAACGTGACAATTGACCTTTCCCAAATCACAACGTCA 1320
Egg      1201 ATTGACTCAAGTATATGCAAGGAAGTTGTCTTAGAGAGTGTGCAGCTTAGGTTAGAAGGAAAGCTGTCATCCCAATACTTCTCCAACGTGACAATTGACCTTTCCCAAATCACAACGTCA 1320
1st Cell 1201 ATTGACTCAAGTATATGCAAGGAAGTTGTCTTAGAGAGTGTGCAGCTTAGGTTAGAAGGAAAGCTGTCATCCCAATACTTCTCCAACGTGACAATTGACCTTTCCCAAATCACAACGTCA 1320
2nd Cell 1201 ATTGACTCAAGTATATGCAAGGAAGTTGTCTTAGAGAGTGTGCAGCTTAGGTTAGAAGGAAAGCTGTCATCCCAATACTTCTCCAACGTGACAATTGACCTTTCCCAAATCACAACGTCA 1320
3rd Cell 1201 ATTGACTCAAGTATATGCAAGGAAGTTGTCTTAGAGAGTGTGCAGCTTAGGTTAGAAGGAAAGCTGTCATCCCAATACTTCTCCAACGTGACAATTGACCTTTCCCAAATCACAACGTCA 1320

Plasmid  1321 GGGTCGCTGGATATAAGCAGTGAAATTGGTAGCATTAACAACACAGTTAATCGGGTCGACGAGTTAATCAAGGAATCCAACGAGTGGCTGAACGCTGTGAACCCCCGCCTTGTGAACAAT 1440
Egg      1321 GGGTCGCTGGATATAAGCAGTGAAATTGGTAGCATTAACAACACAGTTAATCGGGTCGACGAGTTAATCAAGGAATCCAACGAGTGGCTGAACGCTGTGAACCCCCGCCTTGTGAACAAT 1440
1st Cell 1321 GGGTCGCTGGATATAAGCAGTGAAATTGGTAGCATTAACAACACAGTTAATCGGGTCGACGAGTTAATCAAGGAATCCAACGAGTGGCTGAACGCTGTGAACCCCCGCCTTGTGAACAAT 1440
2nd Cell 1321 GGGTCGCTGGATATAAGCAGTGAAATTGGTAGCATTAACAACACAGTTAATCGGGTCGACGAGTTAATCAAGGAATCCAACGAGTGGCTGAACGCTGTGAACCCCCGCCTTGTGAACAAT 1440
3rd Cell 1321 GGGTCGCTGGATATAAGCAGTGAAATTGGTAGCATTAACAACACAGTTAATCGGGTCGACGAGTTAATCAAGGAATCCAACGAGTGGCTGAACGCTGTGAACCCCCGCCTTGTGAACAAT 1440

Plasmid  1441 ACGAGCATCGTAGTCCTCTGTGTCCTTGCCGCCCTGATTATTGTCTGGCTAATAGCGCTGACAGTATGCTTCTGTTACTCCGCAAGATACTCAGCTAAGTCAAAACAGATGAGGGGCGCT 1560
Egg      1441 ACGAGCATCGTAGTCCTCTGTGTCCTTGCCGCCCTGATTATTGTCTGGCTAATAGCGCTGACAGTATGCTTCTGTTACTCCGCAAGATACTCAGCTAAGTCAAAACAGATGAGGGGCGCT 1560
1st Cell 1441 ACGAGCATCGTAGTCCTCTGTGTCCTTGCCGCCCTGATTATTGTCTGGCTAATAGCGCTGACAGTATGCTTCTGTTACTCCGCAAGATACTCAGCTAAGTCAAAACAGATGAGGGGCGCT 1560
2nd Cell 1441 ACGAGCATCGTAGTCCTCTGTGTCCTTGCCGCCCTGATTATTGTCTGGCTAATAGCGCTGACAGTATGCTTCTGTTACTCCGCATGATACTCAGCTAAGTCAAAACAGATGAGGGGCGCT 1560
3rd Cell 1441 ACGAGCATCGTAGTCCTCTGTGTCCTTGCCGCCCTGATTATTGTCTGGCTAATAGCGCTGACAGTATGCTTCTGTTACTCCGCATGATACTCAGCTAAGTCAAAACAGATGAGGGGCGCT 1560

Plasmid  1561 ATGACAGGGATCGATAGTCCATATGTAATACAGAGTGCAACTAAGATGTAG 1611
Egg      1561 ATGACAGGGATCGATAGTCCATATGTAATACAGAGTGCAACTAAGATGTAG 1611
1st Cell 1561 ATGACAGGGATCGATAGTCCATATGTAATACAGAGTGCAACTAAGATGTAG 1611
2nd Cell 1561 ATGACAGGGATCGATAGTCCATATGTAATACAGAGTGCAACTAAGATGTAG 1611
3rd Cell 1561 ATGACAGGGATCGATAGTCCATATGTAATACAGAGTGCAACTAAGATGTAG 1611
```

Plasmid : Plasmids for rescue experiments
Egg : Viruses were propagated in embryonated eggs
1st Cell :
2nd Cell : Each passage viruses in HEp-2 cells
3rd Cell :

rNDV-2F/2HN (APMV-2 HN gene)

```
Plasmid     1 ATGGATTTCCCATCTAGGGAGAACCTGGCAGCAGGTGACATATCGGGGCGGAAGACTTGGAGATTACTGTTCCGGATCCTCACATTGAGCATAGGTGTGGTCTGTCTTGCCATCAATATT 120
Egg         1 ATGGATTTCCCATCTAGGGAGAACCTGGCAGCAGGTGACATATCGGGGCGGAAGACTTGGAGATTACTGTTCCGGATCCTCACATTGAGCATAGGTGTGGTCTGTCTTGCCATCAATATT 120
1st Cell    1 ATGGATTTCCCATCTAGGGAGAACCTGGCAGCAGGTGACATATCGGGGCGGAAGACTTGGAGATTACTGTTCCGGATCCTCACATTGAGCATAGGTGTGGTCTGTCTTGCCATCAATATT 120
2nd Cell    1 ATGGATTTCCCATCTAGGGAGAACCTGGCAGCAGGTGACATATCGGGGCGGAAGACTTGTAGATTACTGTTCCGGATCCTCACATTGAGCATAGGTGTGGTCTGTCTTGCCATCAATATT 120
3rd Cell    1 ATGGATTTCCCATCTAGGGAGAACCTGGCAGCAGGTGACATATCGGGGCGGAAGACTTGGAGATTACTGTTCCGGATCCTCACATTGAGCATAGGTGTGGTCTGTCTTGCCATCAATATT 120

Plasmid   121 GCCACAATTGCAAAATTGGATCACCTGGATAACATGGCTTCGAACACATGGACAACAACTGAGGCTGACCGTGTGATATCTAGCATCACGACTCCGCTCAAAGTCCCTGTCAACCAGATT 240
Egg       121 GCCACAATTGCAAAATTGGATCACCTGGATAACATGGCTTCGAACACATGGACAACAACTGAGGCTGACCGTGTGATATCTAGCATCACGACTCCGCTCAAAGTCCCTGTCAACCAGATT 240
1st Cell  121 GCCACAATTGCAAAATTGGATCACCTGGATAACATGGCTTCGAACACATGGACAACAACTGAGGCTGACCGTGTGATATCTAGCATCACGACTCCGCTCAAAGTCCCTGTCAACCAGATT 240
2nd Cell  121 GCCACAATTGCAAAATTGGATCACCTGGATAACATGGCTTCGAACACATGGACAACAACTGAGGCTGACCGTGTGATATCTAGCATCACGACTCCGCTCAAAGTCCCTGTCAACCAGATT 240
3rd Cell  121 GCCACAATTGCAAAATTGGATCACCTGGATAACATGGCTTCGAACACATGGACAACAACTGAGGCTGACCGTGTGATATCTAGCATCACGACTCCGCTCAAAGTCCCTGTCAACCAGATT 240

Plasmid   241 AATGACATGTTTCGGATTGTAGCGCTTGACCTACCTCTGCAGATGACATCATTACAGAAAGAAACAGCATCCCAAGTCGGGTTCTTGGCTGAAAGTATCAACAATGTTTTATCCAAGAAT 360
Egg       241 AATGACATGTTTCGGATTGTAGCGCTTGACCTACCTCTGCAGATGACATCATTACAGAAAGAAACAGCATCCCAAGTCGGGTTCTTGGCTGAAAGTATCAACAATGTTTTATCCAAGAAT 360
1st Cell  241 AATGACATGTTTCGGATTGTAGCGCTTGACCTACCTCTGCAGATGACATCATTACAGAAAGAAACAGCATCCCAAGTCGGGTTCTTGGCTGAAAGTATCAACAATGTTTTATCCAAGAAT 360
2nd Cell  241 AATGACATGTTTCGGATTGTAGCGCTTGACCTACCTCTGCAGATGACATCATTACAGAAAGAAACAGCATCCCAAGTCGGGTTCTTGGCTGAAAGTATCAACAATGTTTTATCCAAGAAT 360
3rd Cell  241 AATGACATGTTTCGGATTGTAGCGCTTGACCTACCTCTGCAGATGACATCATTACAGAAAGAAACAGCATCCCAAGTCGGGTTCTTGGCTGAAAGTATCAACAATGTTTTATCCAAGAAT 360

Plasmid   361 GGATCTGCAGGCCTGGTTCTTGTTAATGACCCTGAATATGCAGGGGGGATCGCTGTCAGCTTGTACCAAGGAGATGCATCTGCAGGCCTAAATTTCCAGCCCATTTCTTTAATAGAACAT 480
Egg       361 GGATCTGCAGGCCTGGTTCTTGTTAATGACCCTGAATATGCAGGGGGGATCGCTGTCAGCTTGTACCAAGGAGATGCATCTGCAGGCCTAAATTTCCAGCCCATTTCTTTAATAGAACAT 480
1st Cell  361 GGATCTGCAGGCCTGGTTCTTGTTAATGACCCTGAATATGCAGGGGGGATCGCTGTCAGCTTGTACCAAGGAGATGCATCTGCAGGCCTAAATTTCCAGCCCATTTCTTTAATAGAACAT 480
2nd Cell  361 GGATCTGCAGGCCTGGTTCTTGTTAATGACCCTGAATATGCAGGGGGGATCGCTGTCAGCTTGTACCAAGGAGATGCATCTGCAGGCCTAAATTTCCAGCCCATTTCTTTAATAGAACAT 480
3rd Cell  361 GGATCTGCAGGCCTGGTTCTTGTTAATGACCCTGAATATGCAGGGGGGATCGCTGTCAGCTTGTACCAAGGAGATGCATCTGCAGGCCTAAATTTCCAGCCCATTTCTTTAATAGAACAT 480

Plasmid   481 CCAAGTTTTGTCCCTGGTCCTACTACTGCTAAGGGCTGTATAAGGATCCCGACCTTCCATATGGGCCCTTCACATTGGTGTTACTCACATAACATCATTGCATCAGGTTGCCAGGATGCG 600
Egg       481 CCAAGTTTTGTCCCTGGTCCTACTACTGCTAAGGGCTGTATAAGGATCCCGACCTTCCATATGGGCCCTTCACATTGGTGTTACTCACATAACATCATTGCATCAGGTTGCCAGGATGCG 600
1st Cell  481 CCAAGTTTTGTCCCTGGTCCTACTACTGCTAAGGGCTGTATAAGGATCCCGACCTTCCATATGGGCCCTTCACATTGGTGTTACTCACATAACATCATTGCATCAGGTTGCCAGGATGCG 600
2nd Cell  481 CCAAGTTTTGTCCCTGGTCCTACTACTGCTAAGGGCTGTATAAGGATCCCGACCTTCCATATGGGCCCTTCACATTGGTGTTACTCACATAACATCATTGCATCAGGTTGCCAGGATGCG 600
3rd Cell  481 CCAAGTTTTGTCCCTGGTCCTACTACTGCTAAGGGCTGTATAAGGATCCCGACCTTCCATATGGGCCCTTCACATTGGTGTTACTCACATAACATCATTGCATCAGGTTGCCAGGATGCG 600

Plasmid   601 AGCCACTCCAGTATGTATATCTCTCTGGGGGTGCTGAAAGCATCGCAGACCGGGTCGCCTATCTTCTTGACAACGGCCAGCCAGCTCGTGGATGACAACATCAACCGGAAGTCATGCAGC 720
Egg       601 AGCCACTCCAGTATGTATATCTCTCTGGGGGTGCTGAAAGCATCGCAGACCGGGTCGCCTATCTTCTTGACAACGGCCAGCCAGCTCGTGGATGACAACATCAACCGGAAGTCATGCAGC 720
1st Cell  601 AGCCACTCCAGTATGTATATCTCTCTGGGGGTGCTGAAAGCATCGCAGACCGGGTCGCCTATCTTCTTGACAACGGCCAGCCAGCTCGTGGATGACAACATCAACCGGAAGTCATGCAGC 720
2nd Cell  601 AGCCACTCCAGTATGTATATCTCTCTGGGGGTGCTGAAAGCATCGCAGACCGGGTCGCCTATCTTCTTGACAACGGCCAGCCAGCTCGTGGATGACAACATCAACCGGAAGTCATGCAGC 720
3rd Cell  601 AGCCACTCCAGTATGTATATCTCTCTGGGGGTGCTGAAAGCATCGCAGACCGGGTCGCCTATCTTCTTGACAACGGCCAGCCAGCTCGTGGATGACAACATCAACCGGAAGTCATGCAGC 720

Plasmid   721 ATCGTAGCCTCAAAATACGGTTGTGATATCCTATGCAGTATTGTGATTGAAACAGAGGATGAGGATTATAGGTCTGATCCGGCTACTAGCATGATTATAGGTAGGCTGTTCTTCAACGGG 840
Egg       721 ATCGTAGCCTCAAAATACGGTTGTGATATCCTATGCAGTATTGTGATTGAAACAGAGGATGAGGATTATAGGTCTGATCCGGCTACTAGCATGATTATAGGTAGGCTGTTCTTCAACGGG 840
1st Cell  721 ATCGTAGCCTCAAAATACGGTTGTGATATCCTATGCAGTATTGTGATTGAAACAGAGGATGAGGATTATAGGTCTGATCCGGCTACTAGCATGATTATAGGTAGGCTGTTCTTCAACGGG 840
2nd Cell  721 ATCGTAGCCTCAAAATACGGTTGTGATATCCTATGCAGTATTGTGATTGAAACAGAGGATGAGGATTATAGGTCTGATCCGGCTACTAGCATGATTATAGGTAGGCTGTTCTTCAACGGG 840
3rd Cell  721 ATCGTAGCCTCAAAATACGGTTGTGATATCCTATGCAGTATTGTGATTGAAACAGAGGATGAGGATTATAGGTCTGATCCGGCTACTAGCATGATTATAGGTAGGCTGTTCTTCAACGGG 840

Plasmid   841 TCATACACAGAGAGCAAGATTAACACAGGGTCCATCTTCAGTCTATTCTCTGCTAACTACCCTGCGGTGGGGTCGGGTATTGTAGTCGGGGATGAAGCCGCATTCCCAATATATGGTGGG 960
Egg       841 TCATACACAGAGAGCAAGATTAACACAGGGTCCATCTTCAGTCTATTCTCTGCTAACTACCCTGCGGTGGGGTCGGGTATTGTAGTCGGGGATGAAGCCGCATTCCCAATATATGGTGGG 960
1st Cell  841 TCATACACAGAGAGCAAGATTAACACAGGGTCCATCTTCAGTCTATTCTCTGCTAACTACCCTGCGGTGGGGTCGGGTATTGTAGTCGGGGATGAAGCCGCATTCCCAATATATGGTGGG 960
2nd Cell  841 TCATACACAGAGAGCAAGATTAACACAGGGTCCATCTTCAGTCTATTCTCTGCTAACTACCCTGCGGTGGGGTCGGGTATTGTAGTCGGGGATGAAGCCGCATTCCCAATATATGGTGGG 960
3rd Cell  841 TCATACACAGAGAGCAAGATTAACACAGGGTCCATCTTCAGTCTATTCTCTGCTAACTACCCTGCGGTGGGGTCGGGTATTGTAGTCGGGGATGAAGCCGCATTCCCAATATATGGTGGG 960

Plasmid   961 GTCAAGCAGAACACATGGTTGTTCAACCAGCTCAAGGATTTTGGTTACTTCACCCATAATGATGTGTACAAGTGCAATCGGACTGATATACAGCAAACTATCCTGGATGCATACAGGCCA 1080
Egg       961 GTCAAGCAGAACACATGGTTGTTCAACCAGCTCAAGGATTTTGGTTACTTCACCCATAATGATGTGTACAAGTGCAATCGGACTGATATACAGCAAACTATCCTGGATGCATACAGGCCA 1080
1st Cell  961 GTCAAGCAGAACACATGGTTGTTCAACCAGCTCAAGGATTTTGGTTACTTCACCCATAATGATGTGTACAAGTGCAATCGGACTGATATACAGCAAACTATCCTGGATGCATACAGGCCA 1080
2nd Cell  961 GTCAAGCAGAACACATGGTTGTTCAACCAGCTCAAGGATTTTGGTTACTTCACCCATAATGATGTGTACAAGTGCAATCGGACTGATATACAGCAAACTATCCTGGATGCATACAGGCCA 1080
3rd Cell  961 GTCAAGCAGAACACATGGTTGTTCAACCAGCTCAAGGATTTTGGTTACTTCACCCATAATGATGTGTACAAGTGCAATCGGACTGATATACAGCAAACTATCCTGGATGCATACAGGCCA 1080

Plasmid  1081 CCTAAAATCTCAGGAAGGTTATGGGTACAAGGCATCCTATTGTGCCCAGTTTCACTGAGACCTGATCCTGGCTGTCGCTTAAAGGTGTTCAATACCAGCAATGTGATGATGGGGGCAGAA 1200
Egg      1081 CCTAAAATCTCAGGAAGGTTATGGGTACAAGGCATCCTATTGTGCCCAGTTTCACTGAGACCTGATCCTGGCTGTCGCTTAAAGGTGTTCAATACCAGCAATGTGATGATGGGGGCAGAA 1200
1st Cell 1081 CCTAAAATCTCAGGAAGGTTATGGGTACAAGGCATCCTATTGTGCCCAGTTTCACTGAGACCTGATCCTGGCTGTCGCTTAAAGGTGTTCAATACCAGCAATGTGATGATGGGGGCAGAA 1200
2nd Cell 1081 CCTAAAATCTCAGGAAGGTTATGGGTACAAGGCATCCTATTGTGCCCAGTTTCACTGAGACCTGATCCTGGCTGTCGCTTAAAGGTGTTCAATACCAGCAATGTGATGATGGGGGCAGAA 1200
3rd Cell 1081 CCTAAAATCTCAGGAAGGTTATGGGTACAAGGCATCCTATTGTGCCCAGTTTCACTGAGACCTGATCCTGGCTGTCGCTTAAAGGTGTTCAATACCAGCAATGTGATGATGGGGGCAGAA 1200

Plasmid  1201 GCGAGGTTGATCCAAGTAGGCTCAACCGTGTATCTATACCAACGCTCATCCTCATGGTGGGTGGTAGGACTGACTTACAAATTAGATGTGTCAGAAATAACTTCACAGACAGGTAACACA 1320
Egg      1201 GCGAGGTTGATCCAAGTAGGCTCAACCGTGTATCTATACCAACGCTCATCCTCATGGTGGGTGGTAGGACTGACTTACAAATTAGATGTGTCAGAAATAACTTCACAGACAGGTAACACA 1320
1st Cell 1201 GCGAGGTTGATCCAAGTAGGCTCAACCGTGTATCTATACCAACGCTCATCCTCATGGTGGGTGGTAGGACTGACTTACAAATTAGATGTGTCAGAAATAACTTCACAGACAGGTAACACA 1320
2nd Cell 1201 GCGAGGTTGATCCAAGTAGGCTCAACCGTGTATCTATACCAACGCTCATCCTCATGGTGGGTGGTAGGACTGACTTACAAATTAGATGTGTCAGAAATAACTTCACAGACAGGTAACACA 1320
3rd Cell 1201 GCGAGGTTGATCCAAGTAGGCTCAACCGTGTATCTATACCAACGCTCATCCTCATGGTGGGTGGTAGGACTGACTTACAAATTAGATGTGTCAGAAATAACTTCACAGACAGGTAACACA 1320

Plasmid  1321 CTCAACCATGTAGACCCCATTGCCCATACAAAGTTCCCAAGACCATCTTTCGGGCGAGATGCGTGTGCGAGGCCAAACATATGCCCTGCTGTCTGTGTCTCCGGAGTTTATCAGGACATT 1440
Egg      1321 CTCAACCATGTAGACCCCATTGCCCATACAAAGTTCCCAAGACCATCTTTCGGGCGAGATGCGTGTGCGAGGCCAAACATATGCCCTGCTGTCTGTGTCTCCGGAGTTTATCAGGACATT 1440
1st Cell 1321 CTCAACCATGTAGACCCCATTGCCCATACAAAGTTCCCAAGACCATCTTTCGGGCGAGATGCGTGTGCGAGGCCAAACATATGCCCTGCTGTCTGTGTCTCCGGAGTTTATCAGGACATT 1440
2nd Cell 1321 CTCAACCATGTAGACCCCATTGCCCATACAAAGTTCCCAAGACCATCTTTCGGGCGAGATGCGTGTGCGAGGCCAAACATATGCCCTGCTGTCTGTGTCTCCGGAGTTTATCAGGACATT 1440
3rd Cell 1321 CTCAACCATGTAGACCCCATTGCCCATACAAAGTTCCCAAGACCATCTTTCGGGCGAGATGCGTGTGCGAGGCCAAACATATGCCCTGCTGTCTGTGTCTCCGGAGTTTATCAGGACATT 1440

Plasmid  1441 TGGCCGATCAGTACAGCCACCAATAACAGCAACATTGTGTGGGTTGGACAGTACTTAGAAGCATTCTATTCCAGGAAATACCCAAGAATAGGGATAGCAACCCAGTATGAGTGGAAAGTC 1560
Egg      1441 TGGCCGATCAGTACAGCCACCAATAACAGCAACATTGTGTGGGTTGGACAGTACTTAGAAGCATTCTATTCCAGGAAATACCCAAGAATAGGGATAGCAACCCAGTATGAGTGGAAAGTC 1560
1st Cell 1441 TGGCCGATCAGTACAGCCACCAATAACAGCAACATTGTGTGGGTTGGACAGTACTTAGAAGCATTCTATTCCAGGAAATACCCAAGAATAGGGATAGCAACCCAGTATGAGTGGAAAGTC 1560
2nd Cell 1441 TGGCCGATCAGTACAGCCACCAATAACAGCAACATTGTGTGGGTTGGACAGTACTTAGAAGCATTCTATTCCAGGAAATACCCAAGAATAGGGATAGCAACCCAGTATGAGTGGAAAGTC 1560
3rd Cell 1441 TGGCCGATCAGTACAGCCACCAATAACAGCAACATTGTGTGGGTTGGACAGTACTTAGAAGCATTCTATTCCAGGAAATACCCAAGAATAGGGATAGCAACCCAGTATGAGTGGAAAGTC 1560

Plasmid  1561 ACCAACCAGCTGTTCAATTCGAATACTGAGGGAGGGTACTCAACCACAACATGCTTCCGGAACACCAAACGGGACAAGGCATATTGTGTAGTGATATCAGAGTACGCTGATGGGGTGTTC 1680
Egg      1561 ACCAACCAGCTGTTCAATTCGAATACTGAGGGAGGGTACTCAACCACAACATGCTTCCGGAACACCAAACGGGACAAGGCATATTGTGTAGTGATATCAGAGTACGCTGATGGGGTGTTC 1680
1st Cell 1561 ACCAACCAGCTGTTCAATTCGAATACTGAGGGAGGGTACTCAACCACAACATGCTTCCGGAACACCAAACGGGACAAGGCATATTGTGTAGTGATATCAGAGTACGCTGATGGGGTGTTC 1680
2nd Cell 1561 ACCAACCAGCTGTTCAATTCGAATACTGAGGGAGGGTACTCAACCACAACATGCTTCCGGAACACCAAACGGGACAAGGCATATTGTGTAGTGATATCAGAGTACGCTGATGGGGTGTTC 1680
3rd Cell 1561 ACCAACCAGCTGTTCAATTCGAATACTGAGGGAGGGTACTCAACCACAACATGCTTCCGGAACACCAAACGGGACAAGGCATATTGTGTAGTGATATCAGAGTACGCTGATGGGGTGTTC 1680

Plasmid  1681 GGATCATACAGGATCGTTCCTCAGCTTATAGAGATTAGAACGACCACCGGTAAATCTGAGTGA 1743
Egg      1681 GGATCATACAGGATCGTTCCTCAGCTTATAGAGATTAGAACGACCACCGGTAAATCTGAGTGA 1743
1st Cell 1681 GGATCATACAGGATCGTTCCTCAGCTTATAGAGATTAGAACGACCACCGGTAAATCTGAGTGA 1743
2nd Cell 1681 GGATCATACAGGATCGTTCCTCAGCTTATAGAGATTAGAACGACCACCGGTAAATCTGAGTGA 1743
3rd Cell 1681 GGATCATACAGGATCGTTCCTCAGATTATAGAGATTAGAACGGCCACCGGTAAATCTGAGTGA 1743
```

C

NDV F protein

In this study

```
NDV Hitchner B1(Accetion no.AF375823)    1 MGSRPSTKNPAPMMLTIRVALVLSCICPANSIDGRPLAAAGIVVTGDKAVNIYTSSQTGSIIVKLLPNLPKDKEACAKAPLDAYNRTLTTLLTPLGDSIR 100
NDV Beaudette C(Accetion no.JN872154)    1 MGPRPSTKNPVPMMLTVRVALVLSCICPANSIDGRPLAAAGIVVTGDKAVNIFTSCQTGSIIVKLLPNLSKDKEACAKAPLDAYNRTLTTLLTPLGDSIR 100

NDV Hitchner B1(Accetion no.AF375823)  101 RIQESVTTSGGGRQGRLIGAIIGGVALGVATAAQITAAAALIQAKQNAANILRLKESIAATNEAVHEVTDGLSQLAVAVGKMQQFVNDQFNKTAQELDCI 200
NDV Beaudette C(Accetion no.JN872154)  101 RIQESVTTSGGRRQKRFIGAIIGGVALGVATAAQITAAAALIQPKQNATNILRLKESIAATNEAVHEVTDGLSQLAVAVGKMQQFVNDQFNKTAQELGCI 200

NDV Hitchner B1(Accetion no.AF375823)  201 KIAQQVGVELNLYLTELTTVFGPQITSPALNKLTIQALYNLAGGNMDYLLTKLGIGNNQLSSLIGSGLITGNPILYDSQTQLLGIQVTLPSVGNLNNMRA 300
NDV Beaudette C(Accetion no.JN872154)  201 RIAQQVGVELNLYLTELTTVFGPQITSPALNKLTIQALYNLAGGNMDYLLTKLGVGNNQLSSLIGSGLITGNPILYDSQTQLLGIQVTLPSVGNLNNMRA 300

NDV Hitchner B1(Accetion no.AF375823)  301 TYLETLSVSTTRGFASALVPKVVTQVGSVIEELDTSYCIETDLDLYCTRIVTFPMSPGIYSCLSGNTSACMYSKTEGALTTPYMTIKGSVIANCKMTTCR 400
NDV Beaudette C(Accetion no.JN872154)  301 TYLETLSVSTTRGFASALVPKVVTQVGSVIEELDTSYCIETDLDLYCTRIVTFPMSPGIYSCLSGNTSACMYSKTEGALTTPYMTIKGSVIANCKMTTCR 400

NDV Hitchner B1(Accetion no.AF375823)  401 CVNPPGIISQNYGEAVSLIDKQSCNVLSLGGITLRLSGEFDVTYQKNISIQDSQVIITGNLDISTELGNVNNSISNALNKLEESNRKLDKVNVKLTSTSA 500
NDV Beaudette C(Accetion no.JN872154)  401 CVNPPGIISQNYGEAVSLIDKQSCNVLSLDGITLRLSGEFDATYQKNISIQDSQVIITGNLDISTELGNVNNSISNALNKLEESNSKLDKVNVKLTSTSA 500

NDV Hitchner B1(Accetion no.AF375823)  501 LITYIVLTIISLVFGILSLILACYLMYKQKAQQKTLLWLGNNTLDQMRATTKM                                                553
NDV Beaudette C(Accetion no.JN872154)  501 LITYIVLTIISLVFGILSLVLACYLMYKQKAQQKTLLWLGNNTLDQMRATTKM                                                553
```

Transmembrane   Endodomain

NDV HN protein

In this study

Endodomain   Transmembrane

```
NDV Hitchner B1 (Accetion no.AF375823)    1 MDRAVSQVALENDEREAKNTWRLIFRIAILFLTVVTLAISVASLLYSMGASTPSDLVGIPTRISRAEEKITSTLGSNQDVVDRIYKQVALESPLALLNTE 100
NDV Beaudette C (Accetion no.AF309036)    1 MDRAVSQVALENDEREAKNTWRLIFRIAILLLTVVTLATSVASLVYSMGASTPSDLVGIPTRISRAEEKITSALGSNQDVVDRIYKQVALESPLALLNTE 100

NDV Hitchner B1 (Accetion no.AF375823)  101 TTIMNAITSLSYQINGAANNSGWGAPIHDPDYIGGIGKELIVDDASDVTSFYPSAFQEHLNFIPAPTTGSGCTRIPSFDMSATHYCYTHNVILSGCRDHS 200
NDV Beaudette C (Accetion no.AF309036)  101 TTIMNAITSLSYQINGAANNSGWGAPIHDPDFIGGIGKELIVDDASDVTSFYPSAFQEHLNFIPAPTTGSGCTRIPSFDMSATHYCYTHNVILSGCRDHS 200

NDV Hitchner B1 (Accetion no.AF375823)  201 HSHQYLALGVLRTSATGRVFFSTLRSINLDDTQNRKSCSVSATPLGCDMLCSKATETEEEDYNSAVPTRMVHGRLGFDGQYHEKDLDVTTLFGDWVANYP 300
NDV Beaudette C (Accetion no.AF309036)  201 HSHQYLALGVLRTTATGRIFFSTLRSISLDDTQNRKSCSVSATPLGCDMLCSKVTETEEEDYNSAVPTLMAHGRLGFDGQYHEKDLDVTTLFEDWVANYP 300

NDV Hitchner B1 (Accetion no.AF375823)  301 GVGGGSFIDSRVWFSVYGGLKPNTPSDTVQEGKYVIYKRYNDTCPDEQDYQIRMAKSSYKPGRFGGKRIQQAILSIKVSTSLGEDPVLTVPPNTVTLMGA 400
NDV Beaudette C (Accetion no.AF309036)  301 GVGGGSFIDGRVWFSVYGGLKPNSPSDTVQEGKYVIYKRYNDTCPDEQDYQIRMAKSSYKPGRFGGKRIQQAILSIKVSTSLGEDPVLTVPPNTVTLMGA 400

NDV Hitchner B1 (Accetion no.AF375823)  401 EGRILTVGTSHFLYQRGSSYFSPALLYPMTVSDKTATLHSPYTFNAFTRPGSIPCQASARCPNSCVTGVYTDPYPLIFYRNHTLRGVFGTMLDGEQARLN 500
NDV Beaudette C (Accetion no.AF309036)  401 EGRILTVGTSHFLYQRGSSYFSPALLYPMTVSNKTATLHSPYTFNAFTRPGSIPCQASARCPNSCVTGVYTDPYPLIFYRNHTLRGVFGTMLDSEQARLN 500

NDV Hitchner B1 (Accetion no.AF375823)  501 PASAVFDSTSRSRITRVSSSSIKAAYTTSTCFKVVKTNKTYCLSIAEISNTLFGEFRIVPLLVEILKDDGVREARSG                        577
NDV Beaudette C (Accetion no.AF309036)  501 PTSAVFDSTSRSRITRVSSSSTKAAYTTSTCFKVVKTNKTYCLSIAEISNTLFGEFRIVPLLVEILKNDGVREARSG                        577
```

APMV-2 F protein

In this study

```
APMV-2 (Accetion no.LC187305)           1 MNQALVILLVSFQLGVALDNSVLAPIGVASAQEWQLAAYTTTLTGTIAVRFIPVLPGNLSTCAQETLQEYNRTVTNILGPLRENLDALLSDFDKPASRFV 100
APMV-2 Yucaipa (Accetion no.EU338414)   1 MNQALVILLVSFQLGVALDNSVLAPIGVASAQEWQLAAYTTTLTGTIAVRFIPVLPGNLSTCAQETLQEYNRTVTNILGPLRENLDALLSDFDKPASRFV 100

APMV-2 (Accetion no.LC187305)         101 GAIIGSVALGVATAAQITAAVALNQAQENARNIWRLKESIKKTNEAVLELKEGLATTAIALDKVQKFINDDIIPQIKDIDCQVVANKLGVYLSLYLTELT 200
APMV-2 Yucaipa (Accetion no.EU338414) 101 GAIIGSVALGVATAAQITAAVALNQAQENARNIWRLKESIKKTNAAVLELKDGLATTAIALDKVQKFINDDIIPQIKDIDCQVVANKLGVYLSLYLTELT 200

APMV-2 (Accetion no.LC187305)         201 TVFGSQITNPALSTLSYQALYSLCGGDMGKLTELIGVNAKDVGSLYEANLITGQIVGYDPELQIILIQVSYPSVSEVTGVRATELVTVSVTTPKGEGQAI 300
APMV-2 Yucaipa (Accetion no.EU338414) 201 TVFGSQITNPALSTLSYQALYSLCGGDMGKLTELIGVNAKDVGSLYEANLITGQIVGYDPELQIILIQVSYPSVSEVTGVRATELVTVSVTTPKGEGQAI 300

APMV-2 (Accetion no.LC187305)         301 VPRYVAQSRVLTEELDVSTCRFSKTTLYCRSILTRPLPTLIASCLSGKYDDCQYTTEIGALSSRFITVNGGVLANCRAIVCKCVSPPHIIPQNDIGSVTV 400
APMV-2 Yucaipa (Accetion no.EU338414) 301 VPRYVAQSRVLTEELDVSTCRFSKTTLYCRSILTRPLPTLIASCLSGKYDDCQYTTEIGALSSRFITVNGGVLANCRAIVCKCVSPPHIIPQNDIGSVTV 400

APMV-2 (Accetion no.LC187305)         401 IDSSICKEVVLESVQLRLEGKLSSQYFSNVTIDLSQITTSGSLDISSEIGSINNTVNRVDELIKESNEWLNAVNPRLVNNTSIVVLCVLAALIIVWLIAI 500
APMV-2 Yucaipa (Accetion no.EU338414) 401 IDSSICKEVVLESVQLRLEGKLSSQYFSNVTIDLSQITTSGSLDISSEIGSINNTVNRVDELIKESNEWLNAVNPRLVNNTSIIVLCVLAALIIVWLIAI 500

APMV-2 (Accetion no.LC187305)         501 TVCFCYSARYSAKSKQMRGAMTGIDNPYVIQSATKM                                                                 536
APMV-2 Yucaipa (Accetion no.EU338414) 501 TVCFCYSARYSAKSKQMRGAMTGIDNPYVIQSATKM                                                                 536
```

Transmembrane

Endodomain

APMV-2 HN protein

Endodomain Transmembrane

In this study

```
APMV-2 (Accetion no.LC187305)           1 MDFPSRENLAAGDISGRKTWRLLFRILTLSIGVVCLAINIATIAKLDHLDNMASNTWTTTEADRVISSITTPLKVPVNQINDMFRIVALDLPLQMTSLQK 100
APMV-2 Yucaipa (Accetion no.EU338414)   1 MDFPSRENLAAGDISGRKTWRLLFRILTLSIGVVCLAINIATIAKLDHLDNMASNTWTTTEADRVISSITTPLKVPVNQINDMFRIVALDLPLQMTSLQK 100

APMV-2 (Accetion no.LC187305)         101 ETASQVGFLAESINNVLSKNGSAGLVLVNDPEYAGGIAVSLYQGDASAGLNFQPISLIEHPSFVPGPTTAKGCIRIPTFHMGPSHWCYSHNIIASGCQDA 200
APMV-2 Yucaipa (Accetion no.EU338414) 101 EITSQVGFLAESINNVLSKNGSAGLVLVNDPEYAGGIAVSLYQGDASAGLNFQPISLIEHPSFVPGPTTAKGCIRIPTFHMGPSHWCYSHNIIASGCQDA 200

APMV-2 (Accetion no.LC187305)         201 SHSSMYISLGVLKASQTGSPIFLTTASQLVDDNINRKSCSIVASKYGCDILCSIVIETEDEDYRSDPATSMIIGRLFFNGSYTESKINTGSIFSLFSANY 300
APMV-2 Yucaipa (Accetion no.EU338414) 201 SHSSMYISLGVLKASQTGSPIFLTTASHLVDDNINRKSCSIVASKYGCDILCSIVIETENEDYRSDPATSMIIGRLFFNGSYTESKINTGSIFSLFSANY 300

APMV-2 (Accetion no.LC187305)         301 PAVGSGIVVGDEAAFPIYGGVKQNTWLFNQLKDFGYFTHNDVYKCNRTDIQQTILDAYRPPKISGRLWVQGILLCPVSLRPDPGCRLKVFNTSNVMMGAE 400
APMV-2 Yucaipa (Accetion no.EU338414) 301 PAVGSGIVVGDEAAFPIYGGVKQNTWLFNQLKDFGYFTHNDVYKCNRTDIQQTILDAYRPPKISGRLWVQGILLCPVSLRPDPGCRLKVFNTSNVMMGAE 400

APMV-2 (Accetion no.LC187305)         401 ARLIQVGSTVYLYQRSSSWWVVGLTYKLDVSEITSQTGNTLNHVDPIAHTKFPRPSFGRDACARPNICPAVCVSGVYQDIWPISTATNNSNIVWVGQYLE 500
APMV-2 Yucaipa (Accetion no.EU338414) 401 ARLIQVGSTVYLYQRSSSWWVVGLTYKLDVSEITSQTGNTLNHVDPIAHTKFPRPSFRRDACARPNICPAVCVSGVYQDIWPISTATNNSNIVWVGQYLE 500

APMV-2 (Accetion no.LC187305)         501 AFYSRKYPRIGIATQYEWKVTNQLFNSNTEGGYSTTTCFRNTKRDKAYCVVISEYADGVFGSYRIVPQLIEIRTTTGKSE                     580
APMV-2 Yucaipa (Accetion no.EU338414) 501 AFYSRKDPRIGIATQYEWKVTNQLFNSNTEGGYSTTTCFRNTKRDKAYCVVISEYADGVFGSYRIVPQLIEIRTTTGKSE                     580
```

Figure S2. Analysis of chimeric NDVs gnome stability.

(A) Summary of sequenced samples. (B) Comparison of gene sequences in each sample. (C) Comparison of gene sequences between the viruses used in each study.

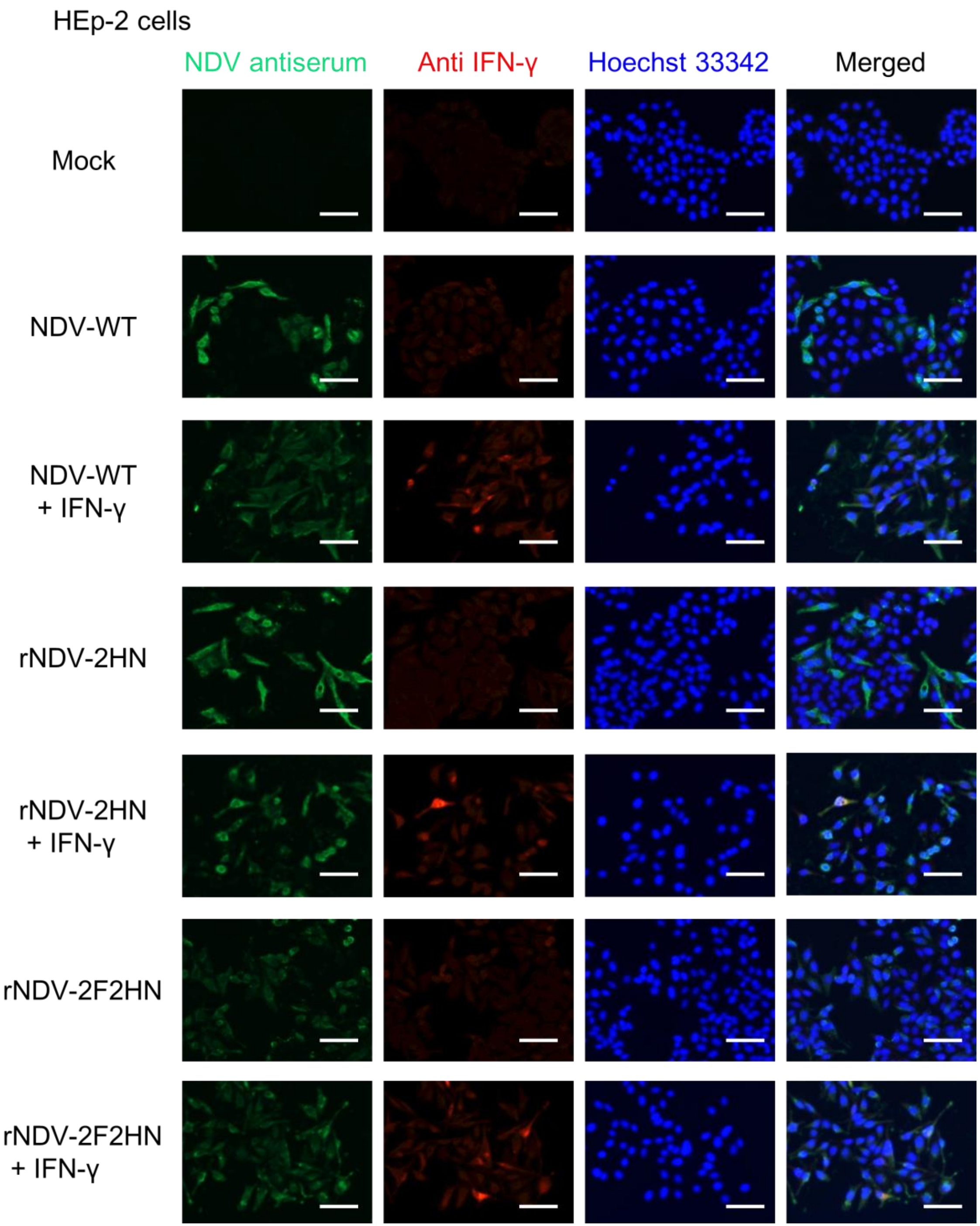
HEp-2 cells
NDV antiserum
Anti IFN-γ
Hoechst 33342
Merged
Mock
NDV-WT
NDV-WT + IFN-γ
rNDV-2HN
rNDV-2HN + IFN-γ
rNDV-2F2HN
rNDV-2F2HN + IFN-γ

Figure S3. INF-γ expression was detected in infected cells with recombinant NDVs + IFN-γ.
Cells infected with each virus at an MOI of 0.5 were stained using anti-NDV antiserum (green), INF-γ antibody (red), and Hoechst 33342 (blue; nuclei). Scale bar: 50 μm

A

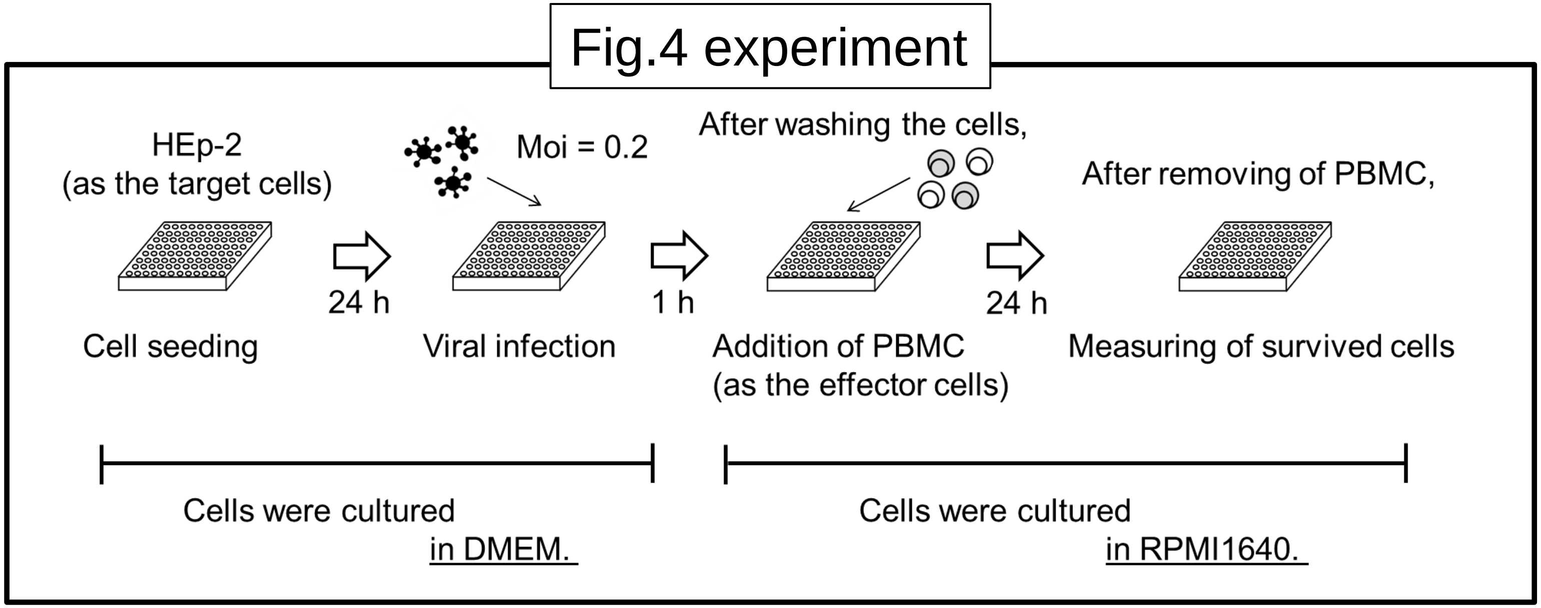

Fig.4 experiment
HEp-2
(as the target cells)
Moi = 0.2
After washing the cells,
After removing of PBMC,
24 h
1 h
24 h
Cell seeding
Viral infection
Addition of PBMC
(as the effector cells)
Measuring of survived cells
Cells were cultured
in DMEM.
Cells were cultured
in RPMI1640.

B

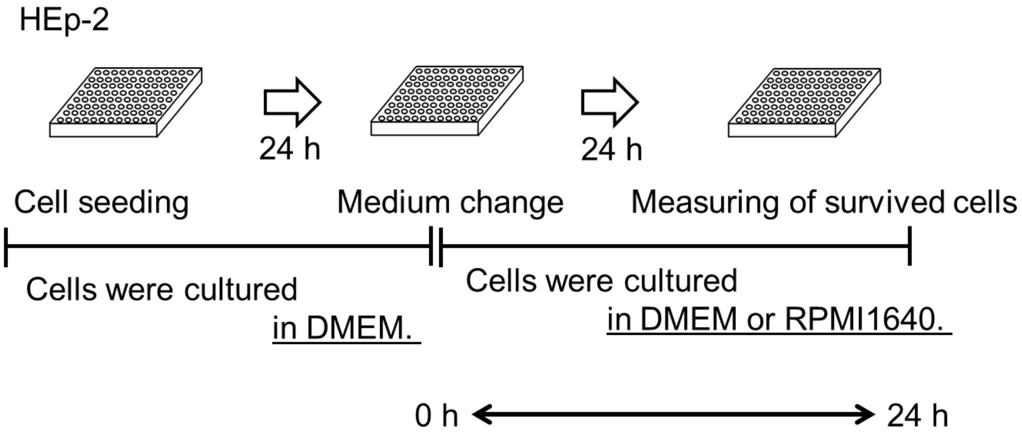

HEp-2
24 h
24 h
Cell seeding
Medium change
Measuring of survived cells
Cells were cultured
in DMEM.
Cells were cultured
in DMEM or RPMI1640.
0 h
24 h

C

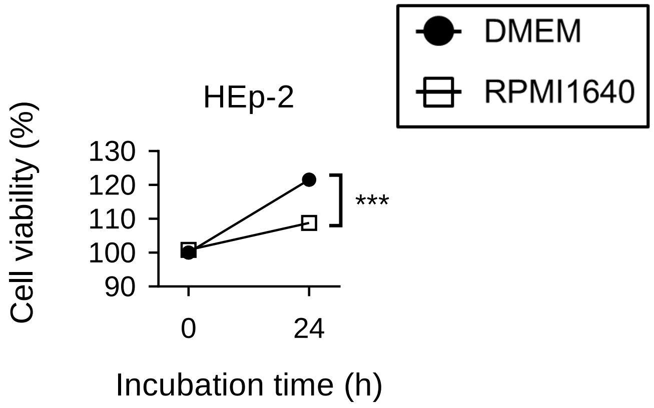

DMEM
RPMI1640
HEp-2
Cell viability (%)
130
120
110
100
90
***
0
24
Incubation time (h)

Figure S4. HEp-2 cells proliferation was modified by RPMI1640 medium.

(A) Schema of the killing assay in Fig. 4. After addition of PBMC, the culture medium was changed to RPMI1640 medium. (B) Schema of the cell viability assay under the killing assay conditions. (C) Cell viability was evaluated by MTT assay at 0 and 24 hours post medium change.

Table S1. Primer sets used in this study.

Primer set	Primer	Sequence
NDV F + NDV HN	Forward	5’-TGACCAGCACATCTGCTCTC-3’
	Reverse	5’-AGCCAAGGTCACTACTGTTA-3’
NDV F + APMV2 HN	Forward	5’-TGACCAGCACATCTGCTCTC-3’
	Reverse	5’-TGATGTCATCTGCAGAGGTA-3’
APMV2 F + APMV2 HN	Forward	5’-ACGACATTGGCTCCGTAACA-3’
	Reverse	5’-TGATGTCATCTGCAGAGGTA-3’
NDV 6001 + NDV 6180	Forward	5’-GAAAATCAAGCGCCTTGC-3’
	Reverse	5’-TCCTTAATGTAGCTAG-3’
